# Spatial transcriptomics reveals influence of microenvironment on intrinsic fates in melanoma therapy resistance

**DOI:** 10.1186/s13059-026-04112-z

**Published:** 2026-05-23

**Authors:** Ryan H. Boe, Catherine G. Triandafillou, Rossana Lazcano, Jennifer A. Wargo, Arjun Raj

**Affiliations:** 1 Genetics and Epigenetics Program, Cell and Molecular Biology Graduate Group, Perelman School of Medicine, University of Pennsylvania, Philadelphia, PA USA; 2 Department of Bioengineering, School of Engineering and Applied Sciences, University of Pennsylvania, Philadelphia, PA USA; 3 Department of Genomic Medicine, Division of Cancer Medicine, The University of Texas MD Anderson Cancer Center, Houston, TX USA; 4 Department of Translational Molecular Pathology, The University of Texas MD Anderson Cancer Center, Houston, TX USA; 5 Department of Genetics, Perelman School of Medicine, University of Pennsylvania, Philadelphia, PA USA

## Abstract

Resistance to cancer therapy is driven by both cell-intrinsic and microenvironmental factors. We use spatial transcriptomics and single-cell RNA sequencing to uncover distinct resistance programs in melanoma cells shaped by intrinsic cellular states and the tumor microenvironment. Consensus non-negative matrix factorization reveals shared intrinsic resistance programs across cell lines. In patient samples, these resistance programs coexist within individual tumors and associate with diverse immune signatures. Single-cell resolution spatial transcriptomics in xenograft models reveals both intrinsically determined and extrinsically influenced resistant fates. This work demonstrates that therapy-resistant fates coexist within distinct microenvironments and that tissue features influence which fate is adopted.

**Supplementary Information:**

The online version contains supplementary material available at 10.1186/s13059-026-04112-z.

## Background

Many aspects of cancer biology, such as oncogenesis, therapy resistance, and metastasis are driven by the behavior of individual cells [1–9]. Often, these outcomes result from mutations in rare cells [10]; however, it has become increasingly clear that non-genetic differences can also be responsible for these phenomena [11]. Recent evidence has shown that these non-genetic differences can come in a spectrum, potentially leading to multiple outcomes as cells transition to becoming, for instance, therapy-resistant [12–16]. The implication is that cell-intrinsic differences can be amplified during the acquisition of therapy resistance. However, it is also clear that in vivo interactions with the local microenvironment, including the immune system, can also shape the outcome of therapy resistance, metastasis, and more. The relative contributions of cell-intrinsic and cell-extrinsic factors to how cells become resistant in vivo remains largely unknown.

Studies of cell-intrinsic factors contributing to therapy resistance have demonstrated that “twin” cells that share the same non-genetic state will typically adopt the same fate when separately subjected to the same external stimulus and that different non-genetic initial states can lead to different outcomes [13, 17–19]. Tracking individual clones longitudinally using DNA barcoding has been essential for connecting initial differences between cells with variability in their final phenotypic characteristics [13, 17–19]. In the context of drug treatment, even genetically identical cancer cells can occupy distinct transcriptional states, which then can dictate their responses to stimuli like drug treatment. Initial work using barcoding [12] showed that these differences are the reason why some cells survive drug and become therapy-resistant while others do not. However, techniques like FateMap, which catalog the variability between therapy-resistant clones, showed that there is further variability between these therapy resistant clones, even if they are all genetically identical. These individual resistant colonies, formed in vitro in homogeneous conditions, display remarkable differences in their morphological, molecular, proliferative, and invasive properties, leading to the conclusion that cell-intrinsic differences can lead to the formation of different resistant “fates” [13]. Critically, these distinct 'fates' represent diverse, stable, non-genetic endpoints characterized by unique molecular signatures and functional properties, despite originating from a seemingly homogeneous population. However, these studies were primarily done in cell culture in highly controlled conditions. It remains to be seen whether these resistant fates appear in the more complex microenvironments that occur in vivo. Furthermore, it is unknown how the complex cell–cell interactions and signaling within the in vivo tumor microenvironment might influence these intrinsically predetermined fate trajectories.

At the same time, it is also clear that, in vivo, there is extrinsic instruction of cell fates via interactions with stromal cells [20, 21], extracellular matrix [22], and cells from the immune system [23, 24]. We currently do not know how these extrinsic cues influence intrinsically specified resistant fates. It is possible, for instance, that different resistant fates can only emerge in different areas of the resistant tumor or that only certain immune cell types are able to infiltrate or otherwise interact with certain resistant fates. Testing for these possibilities requires the use of spatial analysis methods, along with new analytical methods to discriminate between both intrinsic and extrinsic cell fate specification.

**Fig. 1:**
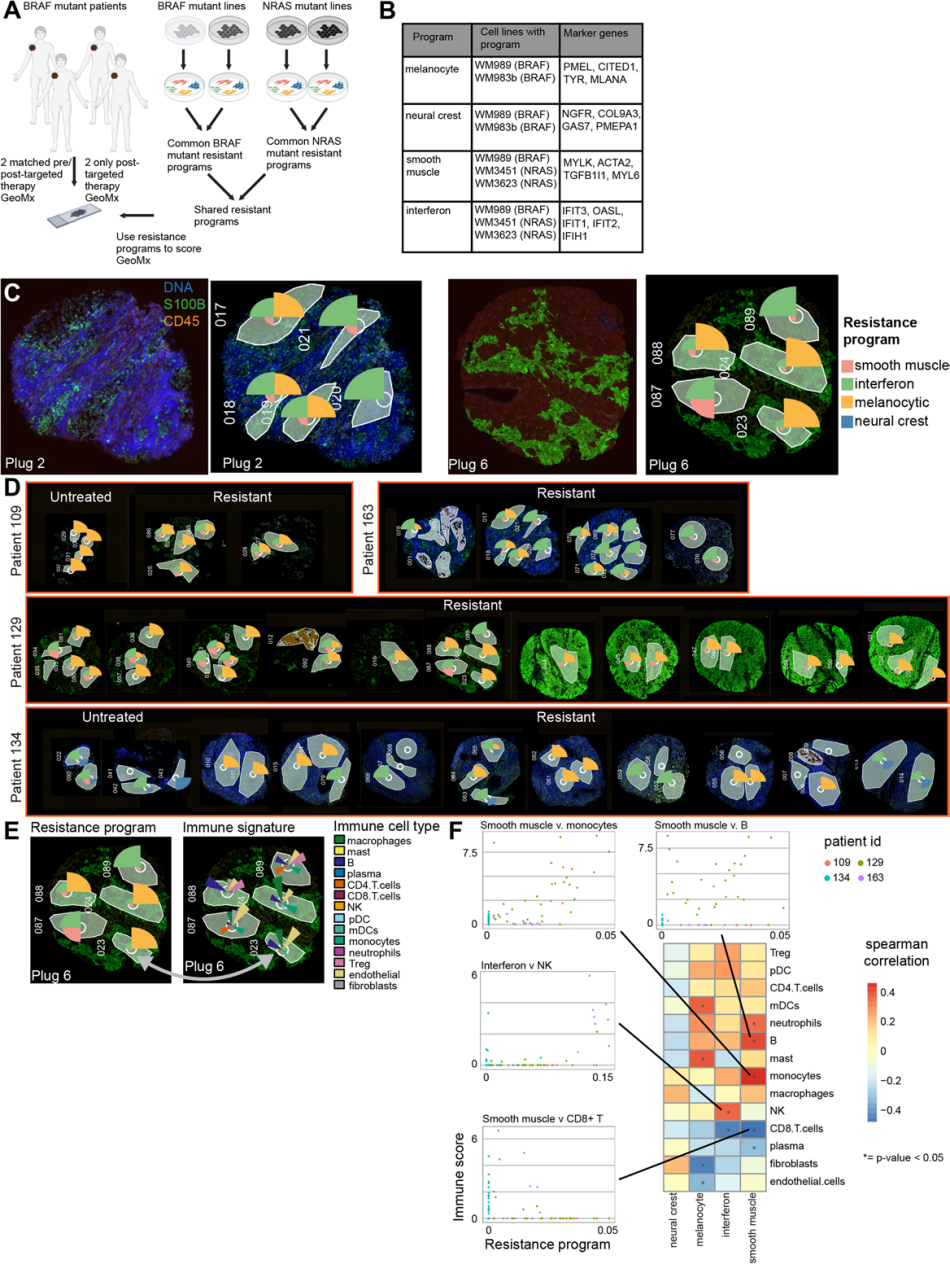
GeoMx spatial transcriptomics reveals distribution of intrinsic melanoma resistance programs and their immune associations in patient samples. **A** Schematic of experimental set-up and computational strategy to identify shared resistance programs. We spatially profiled using the GeoMx system samples from four patients total, two with matched pre-/post-targeted therapy and two with only post-targeted therapy. Separately, we barcoded, treated with targeted therapy, and single-cell sequenced four melanoma cell lines, two with *BRAF* mutations and two with *NRAS* mutations. From each separate single-cell dataset, we found resistance programs using cNMF and then compared them to find shared resistance programs across cell lines and mutations. **B** Table with each intrinsic resistance program, the cell lines that displayed that program, and the top consistent marker genes for the program. **C** Two sets of patient tumor plugs with staining alone (left images) and with region of interest (ROI) and resistance program score drawn (right images). Samples are stained for DNA (SYTO 13, blue), S100B (green), and CD45 (red). For each ROI, the total gene expression profile was deconvolved using a signature matrix of the consensus metaprograms using non-negative least squares. The scores for the four intrinsic resistance programs were normalized to one for each ROI, and the area of each wedge is proportional to its relative resistance program score. **D** Intrinsic resistance program scores for each ROI across all four patients. Intrinsic resistance program scoring is the same as in panel D. **E** Comparison between the melanoma resistance program score and the immune cell signature for each tumor-containing ROI. For each tumor-containing ROI, we estimated the immune cell types present using the SpatialDecon package from NanoString (right image). We can then compare, within each ROI, which resistance programs are expressed (left image) to the immune cell infiltration in the ROI. **F** Heatmap with correlations between resistance program score and immune cell score in resistant sample, tumor-containing ROIs (bottom right) and individual scatter plots for resistance program scores versus immune signature scores (top and left). We calculated the Spearman correlation between the melanoma program score and immune cell score across all post-treatment, tumor ROIs and marked those with *p*-values < 0.05 after Benjamini–Hochberg correction. We pulled scatter plots for four associations that were statistically significant where each dot is a single ROI and is colored by which patient the ROI originated from

Here, we used spatial transcriptomics datasets to look for signatures of diverse resistant fates in patient samples. We integrated multiple in vitro single-cell datasets to arrive at consensus markers of resistant fates and then looked for those fates in therapy-resistant melanoma, finding that these fates exist in patients and that they are correlated with various immune signatures (Fig. 1A). We also analyzed xenograft models of therapy-resistant melanoma with single-cell resolution, finding resistant fates that were intrinsically determined, fates that were extrinsically determined, and fates that had some component of extrinsic and intrinsic determination. Overall, we found that intrinsic melanoma resistance fates are associated with the local microenvironment, suggesting that both intrinsic and extrinsic factors need to be considered when predicting resistant fate specification.

## Results

### Consensus Non-negative Matrix Factorization of FateMap samples reveals shared intrinsic resistance programs

Before analyzing patient samples for the presence of resistant fates, we wanted to systematically define a gene program for each resistant fate. Our strategy leverages previous work using FateMap, a lineage-tracing method designed to dissect cell-intrinsic contributions to fate determination in vitro [13]. FateMap allows us to identify distinct, stable cellular states, or 'fates', that melanoma cells adopt upon acquiring resistance to targeted therapy, and critically, to determine if the specific fate adopted is pre-determined by the cell's state *before* drug exposure. The experimental logic involves: (1) uniquely barcoding clonal *BRAF*V600E mutant melanoma cells, (2) allowing them to divide several times to produce multiple related 'sister' cells sharing the same barcode, (3) physically splitting these sister cells into separate culture wells ('arms'), ensuring they subsequently experience independent microenvironments, and (4) treating both arms with a MAPK inhibitor (vemurafenib or trametinib). After resistance emerges, the resistant cells are transcriptionally profiled (scRNA-seq). By comparing the transcriptional state (resistant fate) of sister cells across the split arms, we can assess intrinsic determination. If sister cells consistently converge on the same resistant fate despite developing resistance independently after the split, it implies that their shared initial state, prior to drug treatment, dictated that outcome. Using this approach, we found five resistant fates–*MLANA* high, *NGFR* high, *ACTA2* high, *IFIT2* high, and *VCAM1* high–that were intrinsically determined. A limited analysis suggested that some number of marker genes for each resistant fate are shared with two *BRAF*^V600E^ lines (WM989 and WM983b) and even in two *NRAS*^Q61K^ lines (WM3251 and WM3623). However, we did not fully assess the similarity between the resistant fates seen in each FateMap sample. We reasoned that resistant fates that recur between multiple cell lines were more likely to reflect general resistant fates that might appear in patient data.

To determine the level of overlap of resistant fates across cell lines, we used consensus non-negative matrix factorization (cNMF) to first find gene expression programs independently for each FateMap sample. cNMF attempts to decompose the single-cell cell-by-gene expression matrix into two new matrices, a program-by-gene signature matrix and a cell-by-program expression matrix [25]. The first matrix gives a number of programs for which each gene has some expression value. For each program, the genes with the top expression, or the top unique expression compared to other programs, can be used to infer the identity of the program. The second matrix gives the amount of each program that each cell expresses. A major advantage of this method compared to traditional clustering methods is that each cell can express multiple programs and this expression is quantitative rather than the single, discrete cluster assignment given by traditional methods. As opposed to clustering, which is often treated as the cell’s “identity,” some programs may capture a cell’s identity, whereas others capture what activity the cell was performing at the time of sequencing. Another advantage of our approach is that by finding gene expression programs independently for each sample and then comparing the programs, we minimize the impact of technical variation and avoid the fraught problem of dataset integration. We checked for recurrent programs across samples by calculating the Jaccard index, which calculates the amount of overlap between two sets of genes, for the top markers for each program and performing hierarchical clustering (Additional file 1: Fig. S1). Our ultimate goal was to identify programs that were represented among multiple FateMap experiments, so we defined a “metaprogram” as a set of cNMF programs that overlap with a Jaccard index of at least 0.1 in at least two FateMap samples, similar to previously defined metaprogram strategies [26, 27]. This analysis yielded 13 expression metaprograms that recurred in at least two FateMap samples. We divided the metaprograms into two categories: “generic” metaprograms that appeared in previous pan-cancer NMF metaprogram analyses and “resistance” metaprograms which corresponded to our previous resistant fates. Reassuringly, we found that a number of the generic metaprograms corresponded to well-defined cellular activities that have been previously reported in pan-cancer NMF analyses [26–28], such as cell division and epithelial-to-mesenchymal transition. In addition, one metaprogram corresponded to high mitochondrial gene expression, which we took to signify damaged and dying cells. Other generic programs included ribosomal protein expression (suggesting active translation), distinct cell division programs (marked by genes involved in M-phase or S-phase respectively), distinct EMT programs (one enriched for canonical markers like *AXL*, another for immediate early genes like *JUNB*/*FOS*), an ECM-related program, a cytoskeleton program, and a program enriched for complement and lysosomal genes (potentially indicating cellular remodeling or stress response). A full list of genes defining each metaprogram is provided in Additional file 2: Table S1.

We found that while many metaprograms were shared between cell lines, others were specific to certain cell lines or to lines that shared a particular driver mutation. From these 13 metaprograms, we recapitulated four of the resistant fates previously identified in FateMap–the melanocyte (*MLANA* high), neural crest (*NGFR* high), smooth muscle (*ACTA2* high), and interferon (*IFIT2* high) metaprograms (Additional file 1: Fig. S2). These specific metaprograms correspond to the non-genetic states shown previously to be intrinsically determined and associated with distinct functional properties, including differences in morphology, proliferation, and invasion in vitro*.* The remaining nine metaprograms were not identified as intrinsically-determined resistance programs in FateMap, which we took to mean they were either cell-activity programs or resistant fates that were not identified in the previous FateMap analysis. The *VCAM1* high fate from FateMap did not appear as a cNMF program in any sample, suggesting that the program may be too weak or too rare to find by this method. The melanocyte and neural crest resistance programs did not occur in the *NRAS* mutant FateMap samples, potentially suggesting that these two resistant fates are specific to *BRAF*^V600E^ mutant melanoma. Alternatively, the lack of the melanocyte and neural crest resistance programs in the *NRAS*^Q61K^ lines may reflect the fact that the *NRAS*^Q61K^ mutant samples were treated with the MEK inhibitor trametinib while the *BRAF*^V600E^ samples were treated with *BRAF*^V600E^ inhibitor vemurafenib, which we have shown decreases the proportion of the *MLANA* high resistant fate [13]. We also found that the smooth muscle and interferon programs were absent from WM983b *BRAF*^V600E^ mutant line.

To assess the robustness and potential generality of these metaprograms beyond our specific cell lines, we examined independent, publicly available scRNA-seq datasets. We identified studies featuring melanoma cell lines, patient-derived xenografts (PDXs), or patient tumor samples treated with targeted MAPK inhibitors [2, 29, 30]. We performed cNMF independently on each of these external datasets to identify their constituent gene expression programs. We first calculated the Jaccard index between each external program and program in our dataset and clustered the data, analogous to how we found metaprograms in the internal data only, yielding at large 154 × 154 heatmap (Additional file 1: Fig. S3). We then compared the externally derived programs with our 13 internal metaprograms by calculating the fraction of genes of each metaprogram that appeared in each external cNMF program. This comparison revealed representation of nearly all our metaprograms within these independent datasets, indicating conservation across different experimental systems (Additional file 1: Fig. S4A). Crucially, we found evidence for the four resistance metaprograms central to our subsequent analyses – the melanocyte, neural crest, smooth muscle, and interferon programs – appearing in various samples across these external studies (Additional file 1: Fig. 4B). This supports the idea that these resistance programs represent conserved biological states relevant to melanoma therapy resistance across different biological contexts.

Next, we took the intersection of the marker genes for each resistance program to identify a consensus marker gene set for each. These are the key marker genes that we used to look for metaprogram expression in patient samples. While many of the genes identified in this manner were the same as those previously identified by single-cell clustering, our method identified unique top markers that were shared across samples, such as *PMEPA1* for the neural crest program, *TGFB1I1* and *MYL6* for the smooth muscle program, *CITED1* and *TYR* for the melanocyte program, and *IFIT3*, *IFIT1*, and *IFIH1* for the interferon program (Fig. 1B). Together, these results show that very different cell lines share some programs, while others are unique to each cell line and that there is a consensus set of marker genes for each resistance program across cell lines. To assess the consistency of these metaprograms, we examined the variability between biological replicates of the WM989 cell line (FateMap experiments FM01 and FM02). While individual gene lists for each metaprogram showed partial overlap between replicates (45–86% overlap based on top 100 genes, Additional file 3: Table S2; Additional file 1: Fig. S5), the overall rank correlation of genes within each program was statistically significant for most metaprograms (Additional file 4: Table S3). Comparing Jaccard indices between biological replicates (mean overlap range 0.29–0.75) and technical replicates (mean overlap range 0.23–0.92) suggested that while technical noise exists, a component of the variability arises from true biological differences between independent experiments (Additional file 1: Fig. S6). However, the concordance between biological replicates remains substantial and is considerably higher than typically observed between different patient tumors [27, 28]. This higher concordance suggests that despite inherent biological and technical variability, the core gene signatures identified represent reproducible biological states within this experimental system.

### Spatial transcriptomics reveals intrinsic resistance programs in patient samples

Having identified a consensus gene set for each metaprogram, we next asked whether there was any evidence for these metaprograms in patient samples using our recently published GeoMx dataset (Additional file 1: Fig. S7). Crucially, our aim was not to define patient-specific programs de novo, but rather to specifically test the hypothesis that the consensus resistance signatures derived from our in vitro experiments are present and spatially organized within in vivo patient tumors. Our previous analysis revealed that several key resistance markers were differentially expressed in different regions of the same tumor; however, we did not quantify expression beyond the level of individual marker genes. We used non-negative least squares deconvolution to quantify the amount of gene metaprogram expression in each region (Fig. 1C). We performed the deconvolution with 12 of the 13 metaprograms (we removed the mitochondrial metaprogram, which represented poor-quality cells) (Additional file 1: Fig. S8). Importantly, since some genes expressed by resistant melanoma cells, including key markers of resistant fates, are also expressed tumor stromal and immune cells, we excluded metaprogram genes that were known to be expressed in cells of the tumor microenvironment (see Methods for details and Additional file 5: Table S4 for metaprogram deconvolution matrix). In addition to deconvolving the resistant samples, we also performed the deconvolution on the pre-treatment samples, even though the metaprograms were derived from resistant cell expression. We determined the goodness of fit of the deconvolution by calculating the log-likelihood of the deconvolution assuming a negative binomial model for the expression of each gene in the deconvolution, where the mean for each gene was the predicted value calculated by multiplying the signature matrix by the transpose of the deconvolution matrix, essentially reversing the deconvolution and assessing how close this predicted value was to the true expression value. We removed instances where the predicted value was zero since a mean of zero is not possible with a negative binomial model. Since we had no estimate of the variance, we used three different values and compared the results. We then calculated a null distribution using a permutation test. We shuffled the row labels (genes) of the signature matrix 1000 times, deconvolved the data, and then performed the log-likelihood calculation described above with the three variance values. We then calculated the total log-likelihood for each region and compared it to the total log-likelihood for each region under the null distribution. We found that our deconvolution was better than shuffled null for 92 out of 95 regions at a *p*-value cutoff of 0.01 (Additional file 6: Table S5). Overall, we were satisfied that the deconvolution was of sufficient quality to look at the distribution of resistance metaprograms in our patient samples.

We decided to focus on the results from the four resistance metaprograms that corresponded to intrinsically determined fates in the original FateMap analysis, the melanocyte, neural crest, smooth muscle, and interferon metaprograms. We found that all but seven of the 92 regions had non-zero resistance program expression, confirming the presence of resistance programs in patient samples (Fig. 1D). We found that while there was a patient-specific signature to resistance program expression, there were also many examples of adjacent regions from the same biopsy plug with very different resistance program expression, suggesting that multiple resistant fates can emerge within patients, albeit with patient-specific biases in the particular resistant fates that do emerge (Fig. 1D). This patient-specific bias can even be seen in pre-treatment samples, with patient 109 showing high melanocyte program scoring pre-treatment while patient 134 showed no melanocyte scoring pre-treatment. The scoring of resistance programs in pre-treatment samples likely reflects some degree of cell state heterogeneity in the drug-naive population, as has been shown by others [31], and that these drug-naive transcriptional programs partially overlap with our resistance programs. However, the fact that different regions of the same tumor display different resistance programs suggests some degree of intrinsic fate determination. It is important to note that these resistant cell fate differences observed in tissue may reflect genetic heterogeneity throughout the tumor; our data show that it is consistent with the non-genetic variability observed in the clonal cell lines originally observed in FateMap. Overall, our analysis suggested that multiple resistant fates emerge within a single patient, although each patient may have a bias towards a particular distribution of these fates.

### Intrinsic resistance programs are associated with the tumor immune microenvironment

We were curious whether we could find evidence of specific interactions between particular resistance programs and the tumor microenvironment. We performed an independent deconvolution analysis on the same GeoMx dataset using a tumor immune signature (Additional file 1: Fig. S9, see Additional file 7: Table S6 for the deconvolution matrix). We therefore were able to compare, for each region, the resistance program score and the immune signature score and look for associations between them (Fig. 1E). We limited our analysis to resistant sample regions that were annotated as containing predominantly tumor cells by a pathologist (Additional file 8: Table S7). Using the Spearman correlation, we found multiple statistically-significant associations between the melanocytic, smooth muscle, and interferon resistance programs and the tumor microenvironment (FDR < 0.05 after correcting for multiple comparisons), though we do note that there were differences among patients and that not all samples exhibit these associations (Fig. 1F). In particular, there was a positive association between interferon program expression and natural killer (NK) cell score, though the association was mostly driven by a single patient (Spearman ρ = 0.36, p = 0.002). We also found that the smooth muscle program was positively associated with B cell infiltration (Spearman ρ = 0.41, p = ​​0.0004) but negatively associated with CD8 + T cell infiltration (Spearman ρ = -0.48, p = 3 × 10^–5^), suggesting that resistant fate may influence or be influenced by the makeup of tumor-infiltrating lymphocytes in a region. The smooth muscle program had, of all the resistant fates, the most positive associations with the enrichment of different immune cell types, suggesting that it may be the most immunogenic. No associations reached statistical significance for the neural crest program, likely due to the small number of regions that contained this program. A statistically significant, positive association with macrophage infiltration emerged if we included pre-treatment regions in the analysis (Spearman ρ = 0.40, p = 0.0003) (Additional file 1: Fig. S10). Since these samples were not strictly independent because they arose from the same patients, we also employed linear mixed-effects modeling using the patient_id as a random intercept. While linear mixed-effects models accounting for patient-level effects yielded partially consistent findings (Additional file 1: Fig. S11), we highlighted these Spearman correlations because they represent visually apparent trends reflecting the rest of the data.

These observed associations, while correlative, may reflect underlying biological interactions. For instance, the positive association between NK cells and the interferon program score could arise from IFN-γ secreted by activated NK cells inducing ISGs (characteristic of the interferon program) in tumor cells, potentially reinforcing an intrinsically programmed state or reflecting an adaptive response to immune engagement. Melanoma cells, in particular, have been shown to upregulate interferon response genes as an adaptive mechanism against immune attack [32, 33]. Similarly, the smooth muscle program, marked by genes involved in TGF-β signaling and ECM remodeling, showed opposing associations with B cells (positive) and CD8 + T cells (negative). This pattern is consistent with the known immunosuppressive effects of TGF-β on cytotoxic T cells and its potential role in B cell responses [34, 35], alongside potential physical effects of ECM changes on T cell infiltration.

### Single-cell spatial transcriptomics reveals intrinsically and extrinsically determined resistant fates

**Fig. 2:**
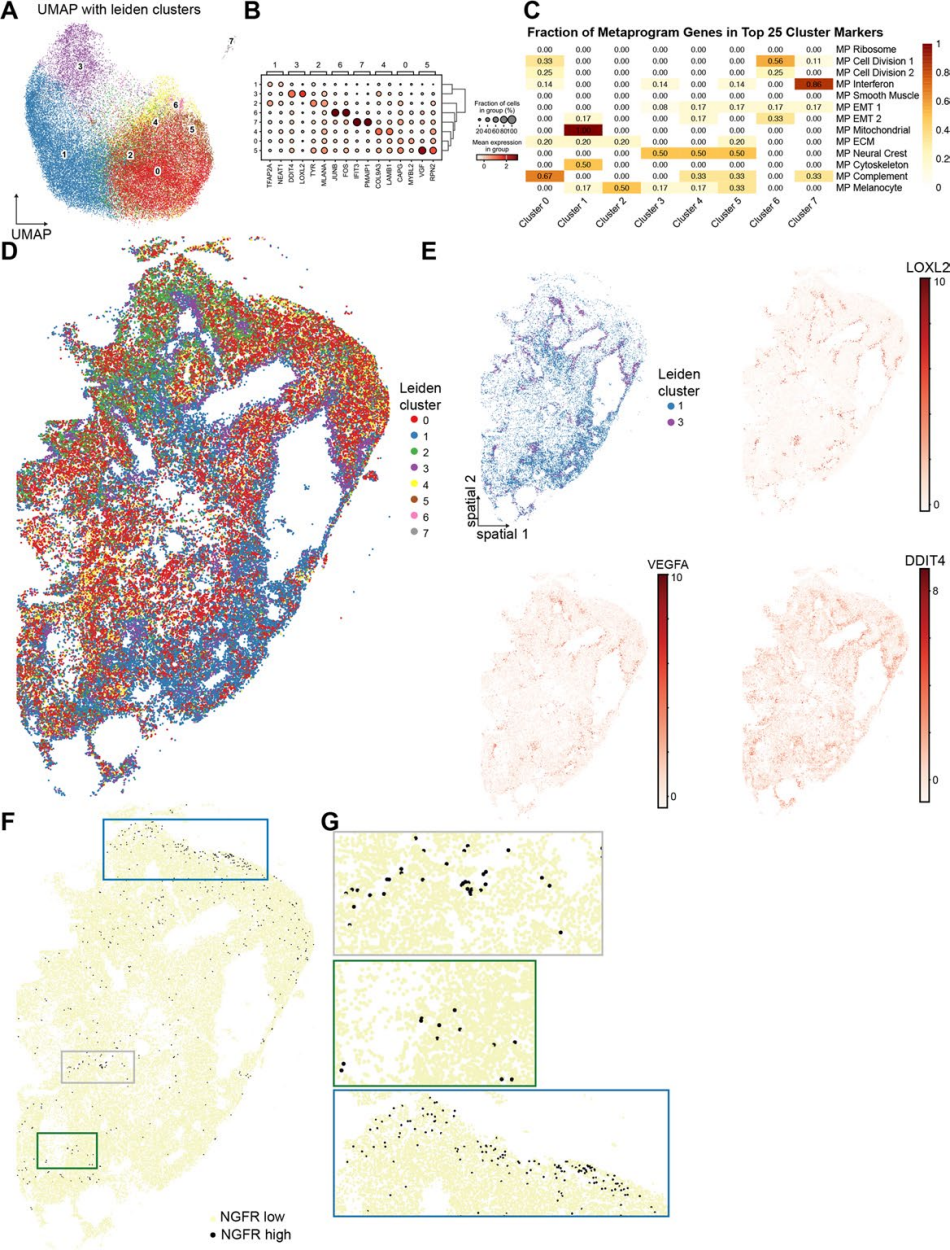
Single-cell spatial transcriptomics of patient-derived xenografts shows intrinsically and extrinsically determined resistant fates. **A** Uniform manifold approximation and projection (UMAP) applied to the first 50 principal components for 51,897 cells. Cells are colored according to leiden clustering using 10 neighbors and a resolution of 0.6. **B** Expression dotplot of the top two marker genes for each cluster across all clusters. Dot size is the fraction of cells in each cluster that express the gene and color is the mean expression of the gene in that cluster. Cluster order on the y-axis is determined by hierarchical clustering. Some leiden clusters have clear, distinct marker genes, such as cluster 3 (DDIT4, LOXL2), cluster 6 (JUNB, FOS), and cluster 7 (IFIT3, PMAIP1). Other clusters, such as cluster 1, have less distinct marker genes. **C** Fraction of genes from each metaprogram in the top 25 markers of each cluster. To assess the overlap between Leiden clusters from single-cell spatial transcriptomics and metaprograms, the top 25 genes by expression were extracted from each Leiden cluster and the fraction of the genes from each metaprogram was calculated, from 0 (white) to 1 (dark red). **D** Spatial plot of 51,897 cells colored by leiden cluster. Clusters 0, 2, 4, 5, 6, and 7 are dispersed throughout the tissue and are intermixed. Clusters 1 and 3 co-occur around areas of likely necrosis in the tissue section. **E** Four spatial plots emphasizing patterns around necrotic regions. Clusters 1 and 3 circumscribe areas of necrosis in the tissue (top left plot). LOXL2, VEGFA, and DDIT4 show expression gradients around the same areas of the tissue (top right, bottom plots). **F** Spatial plot showing distribution of NGFR high cells. Cells were binned as either NGFR high or NGFR low by manual thresholding based on the distribution of normalized NGFR values. NGFR high cells are relatively rare and occur in small clusters. NGFR high cells are enriched towards the tissue section edge. **G** Zoomed-in regions of the spatial plot F. NGFR high cells occur in small clusters, consistent with transient heritability seen in vitro (top, middle). NGFR high cells are not evenly distributed in the tissue and occur more often at the edge of the tissue sample, consistent with some degree of extrinsic fate determination (bottom)

We next wanted to look for spatial patterns of expression in targeted therapy-resistant melanoma at the single-cell level. To do so in a more precisely controlled environment than human samples, we used the Spatial Genomics platform with a custom panel of 427 genes (Additional file 9: Table S8) to spatially profile single cells of clonal *BRAF* mutant WM989 A6-G3 5a3 cells that were implanted into a mouse xenograft and then made resistant via treatment with targeted therapy. Unsupervised clustering of single cells using the leiden algorithm, a community detection algorithm that iteratively assigns cells into “communities” based on connectivity in PCA space, revealed seven clusters (Fig. 2A). Consistent with previous in vivo analysis of similar xenograft models using single-molecule RNA FISH or clampFISH [13, 36], these seven included clusters that clearly mapped to the melanocyte and the interferon resistance program found from in vitro FateMap (Fig. 2B). There additionally was a cluster that expressed *COL9A3* and *S100B* and was weakly enriched for *NGFR*, though these genes were less confined to a single cluster than in vitro (Additional file 1: Fig. S12). Notably, we did not find the smooth muscle state, likely partially because the *ACTA2* probe, a key marker of the state, failed to hybridize for technical reasons. Uniquely, compared to in vitro, we also found a cluster highly enriched for *JUNB and FOS*. This cluster likely corresponds to the “stress-like” state previously described in zebrafish models of melanoma, which also showed high expression of *JUNB* and *FOS* [37] (Fig. 2B). Finally, we found a number of clusters that did not clearly map to a resistance fate (Fig. 2B). Without lineage information, which was available in the in vitro FateMap study 13, discerning whether these represent stable, heritable fates versus transient cell states or clustering artifacts is challenging. To more formally assess the relationship between the Leiden clusters identified in the xenograft and the resistance metaprograms derived from in vitro data, we performed a quantitative overlap analysis. We calculated the fraction of genes defining each metaprogram (limited to those present in our 427-gene spatial panel) that were among the top 25 differentially expressed marker genes for each Leiden cluster (Fig. 2C). This analysis confirmed strong correspondence between cluster 2 and the melanocyte metaprogram, and between cluster 7 and the interferon metaprogram. It also supported the notion that the neural crest metaprogram was distributed across multiple clusters, primarily clusters 3, 4, and 5. Furthermore, this revealed potential associations between cluster 6 (marked by *JUNB*/*FOS*) and cell cycle programs, cluster 1 and mitochondrial/cytoskeleton programs, and cluster 0 and the complement metaprogram, suggesting additional biological states captured by the xenograft clustering beyond the primary resistance fates.

We were curious about the identity and spatial organization of the leiden clusters (clusters 1 and 3) that were specific to the xenograft setting. We first noted that while most cells in the xenograft were high in *MLANA* expression, cells in clusters 1 and 3 were marked by low *MLANA* expression (Fig. 2B). Principal component analysis showed that genes corresponding to melanocyte identity, especially *MLANA*, were the drivers of PC1, and that as visualized in the PCA plot, clusters 1 and 3 have lower values along the PC1 axis (Additional file 1: Fig. S13, left). Consistent with this, expression of MLANA strongly correlates with PC1 (Additional file 1: Fig. S13, right). Individual marker analysis demonstrated that cluster 1 was marked by *TFAP2A* and *NEAT1*, though notably these genes were not very discriminatory for the cluster (Fig. 2B). Instead, its defining characteristic seemed to be its depletion of *MLANA* expression. Cluster 3, in contrast, was strongly marked by *DDIT4* and *LOXL2,* along with low *MLANA* expression (Fig. 2B). We next looked at the spatial distribution of leiden clusters throughout the tissue sample (Fig. 2D) While most clusters were relatively evenly dispersed throughout the tissue, clusters 1 and 3 formed distinct patterns of zonation (Fig. 2D, 2E top left). Clusters 1 and 3 formed relatively large, contiguous areas of expression consisting of dozens to hundreds of individual cells (Fig. 2E, top left). These zonation patterns repeatedly formed around regions with very few to no cells, which we took to be necrotic regions of the tissue. We also found spatial patterns of *DDIT4, VEGFA,* and *LOXL2* expression around these necrotic regions (Fig. 2E, top right, bottom left and right). Overall, the large numbers of cells involved in this patterning, along with a clear spatial cue, suggest that these resistant fates are extrinsically specified.

In contrast, we found that fates that were found to be intrinsically determined in FateMap were more evenly dispersed throughout the bulk of the tissue. Cluster 7, the interferon cluster, was relatively evenly dispersed and formed small patches of 3–4 cells (Additional file 1: Fig. S14A). Since we know that the interferon fate is intrinsically determined in vitro, these small patches of cells presumably came from a single parental cell of origin, though we cannot definitively conclude this because the xenograft lacks lineage barcodes. Cluster 6, which expressed markers of the previously described “stress-like” state, was also evenly dispersed throughout the tissue and occurred in small patches of 3–5 cells (Additional file 1: Fig. S14B). This pattern is suggestive of the stress-like state being similarly intrinsically-determined in the context of a xenograft, though we do not have in vitro barcode data to corroborate. In contrast, the distribution of cells with high expression of *NGFR*, which marks the neural crest resistant fate, showed a mixed distribution in the xenograft (Fig. 2F). *NGFR*^high^ cells were spread throughout the tissue and occurred in small patches of 3–4 cells, but there also were distinct regions that were enriched for *NGFR*^high^ cells, particularly at the edge of the xenograft (Fig. 2F, 2G). The influence of spatial position on the frequency of small patches of *NGFR*^high^ cells suggests that the *NGFR*^high^ fate, though shown to be intrinsically determined in vitro, may also be biased depending on the microenvironmental context. Thus, we were able to find both intrinsically and extrinsically determined fates in our single-cell spatial transcriptomic dataset.

## Discussion

In this work, we show that there exists a consensus set of intrinsic resistance programs that associate with the extrinsic tumor microenvironments in melanoma patients treated with targeted therapy. We further show in xenografts that some resistant fates are determined intrinsically and some extrinsically by features of the tumor microenvironment. These are not exclusive possibilities because some fates appear to be determined intrinsically but with a component of bias based on the external environment. Resistant fate specification in this system thus is a complicated mixture of intrinsic and extrinsic determination and may be different for each specific resistance fate.

A common observation in transcriptomics from patient samples is that individual patient signature is the strongest axis of variation and that patient samples tend to cluster separately from each other, potentially due to technical batch effects or biological patient-specific differences. Our work offers a possible reframing of the problem. If one knows the underlying non-genetic programs in the system, these programs can be effectively thought of as a basis set for each transcriptome. This approach avoids the complicated problem of integration and allows one to instead make comparisons across patients by using the relative amount of each non-genetic program. In our case of resistance programs, we used this line of reasoning to calculate the underlying resistance program sets for each sample and only then make comparisons. Of course, this framework requires one to know the basis set a priori, which is not always possible.

One major outstanding question is how the underlying genetic background of a cancer cell affects the non-genetic states the cell can access. Our clonal cell line evidence suggests that both driver mutation and broader genetic background impact resistant fate specification, and our patient data shows that certain patients are biased toward certain resistant fates. Atlas-scale single-cell sequencing of resistant tumors from many patients, with or without lineage imputation, could begin to conclusively answer how driver mutation or bulk tumor genome interacts with non-genetic fates. However, since tumors are polyclonal, single-cell genomics combined with transcriptomics would be required to understand how intratumoral genetic heterogeneity drives non-genetic heterogeneity.

Conversely, the role of non-genetic heterogeneity when genetic heterogeneity is available is still unclear. One possibility has to do with timescales. In our system, prior to becoming resistant, a slowly fluctuating non-genetic state drives therapy resistance, but then following acquisition of resistance, the resistant states become “locked in” and are stable over many generations but are not the result of genetic mutations. In patient samples, we see the same resistant states but do not know conclusively whether they are maintained by mutation. One model is that, in vivo, the locked-in, non-genetically-encoded states serve as a temporary bridge until the state can be permanently fixed by mutation. It could also be that different mutations allow for different sets of resistant fates to emerge. Such questions could be answered by serial simultaneous profiling of single-cell transcriptomes and genomes.

One limitation of our data is that the direction of causation is impossible to determine. Though we know that in vitro, these resistant fates are intrinsically determined, our in vivo data lacks lineage information. Future advances in lineage barcoding in single-cell spatial transcriptomics may soon overcome this barrier [38]. It may be that differences in the tumor immune microenvironment allow certain resistant fates to emerge and others to die. Or it may be that immune infiltration comes after the resistant fate is determined, with different populations of immune cells attracted to different resistant fates. At least in the context of immunotherapy, non-genetic states can influence T-cell infiltration [39]. In either case, our data clearly demonstrates that resistant fates in targeted therapy interact with the tumor microenvironment.

## Conclusions

This work comprehensively demonstrates that resistance to targeted therapy in melanoma is governed by a complicated interplay between cell-intrinsic resistance programs and the extrinsic tumor microenvironment. By integrating in vitro single-cell sequencing and spatial transcriptomics from patient samples and xenografts, we identified a consensus set of intrinsically determined resistance fates—including melanocyte, neural crest, smooth muscle, and interferon programs—that coexist within individual tumors. Crucially, our spatial analysis in patient tumors revealed that these intrinsic resistance programs associate with distinct immune microenvironments, such as the smooth muscle program's opposing associations with B cells (positive) and CD8 + T cells (negative). Furthermore, single-cell spatial analysis in xenografts showed that resistant fates are specified by a mixture of intrinsic determination and extrinsic influence from tissue features. This study offers a new framework for analyzing patient transcriptomics by considering non-genetic programs as a basis set, fundamentally underscoring that both intrinsic and microenvironmental factors must be accounted for when predicting and treating therapy resistance.

## Methods

### Single-cell RNA-seq preprocessing

For input into the cNMF algorithm, we processed each FateMap experiment separately. For each dataset, we started with the cell-by-gene count matrix from CellRanger. When applicable, we applied the same processing done in the original manuscript. Briefly, we required each gene to be present in at least 3 cells, and each cell to have a minimum of 300 features. Other preprocessing parameters were specific to each dataset. All datasets except for the WM989 biological replicate had two technical replicates. Technical replicates were integrated using Seurat’s SCTransform command with the vars.to.regress parameter set to the technical replicate. For the WM989 biological replicate, we applied SCTransform without regressing out a variable. For each sample, we then extracted the ‘counts’ layer for downstream analysis.

### Consensus non-negative matrix factorization

We performed cNMF analysis as previously described [25]. cNMF takes a cell by gene count matrix and produces a matrix of gene expression programs and a matrix with the usage of each program for each cell. The algorithm runs this factorization, which has a degree of randomness, multiple times and performs a meta-analysis of the factorizations to improve robustness and accuracy. Since the user must manually select the number of programs, we ran the factorization independently on each sample over a range of programs from 5 to 13, per the developer’s recommendation. Following developer recommendations, we evaluated values of k ranging from 5 to 13. The optimal k for each sample was selected manually by inspecting the stability and error plots generated by cNMF across the range of k (Additional file 1: Fig. S15), balancing statistical stability with the biological interpretability and recovery of expected gene programs (e.g., cell cycle, known resistance markers) [25]. We confirmed that the core programs were largely robust to minor variations in k using alluvial plots comparing programs at k-1, k, and k + 1 (Additional file 1: Fig. S16). We finally filtered outlier factorizations and obtained the consensus matrices for each sample.

### Gene expression program overlap

To identify programs that were common across samples, we used a “metaprogram” strategy similar to what has been previously described [26, 27]. We took the top 100 marker genes for each NMF gene expression program based on the z score for that program. We then calculated the Jaccard index between the marker genes across all samples, thus finding the overlap between programs. We then clustered the Jaccard index results and considered a Jaccard index of greater than or equal to 0.1 as a significant overlap. Custers that included at least two samples were called metaprograms. We manually inspected the marker genes for the programs that made up each program to assign the metaprogram identity.

### GeoMx data processing

Sequencing results were downloaded and re-processed using slightly different parameters using the manufacturer’s proprietary software package. Default values were used except where otherwise noted. Segments with fewer than 1000 reads, less than 80% aligned reads, or less than 50% sequencing saturation were excluded from downstream analysis. Probes were excluded if the ratio of the geometric mean of the probe in all segments to the geometric mean of the probe in the target was less than or equal to 0.1 or if the probe failed the Grubbs outlier test in 20% or more of samples. Finally, we kept targets that exceeded a threshold (higher of limit of quantification or count of two) in at least 2% of samples. We exported the filtered count data for downstream analysis. Finally, we had a pathologist manually annotate the hematoxylin and eosin eosin-stained and fluorescent-stained regions for a number of features reported in Additional file 8: Table S7.

### Non-negative least squares metaprogram deconvolution of GeoMx data

To perform non-negative least squares (nnls) deconvolution, we needed to construct a signature matrix and a target matrix in the same units. For the signature matrix, we imported all the genes that were markers for the metaprograms identified above. We then imported the gene usage matrices for each experiment in units of CPM. We filtered these usage matrices to include only the marker genes from the metaprogram. We constructed the signature matrix by taking the mean gene usage across individual programs from each FateMap experiment for each metaprogram. Finally, we filtered genes from the signature matrix that appeared in the safeTME signature matrix in order to minimize potential confounding from genes expressed in the tumor microenvironment (Additional file 5: Table S4). We constructed the target matrix by doing upper quartile normalization and CPM calculation of the filtered GeoMx count data using edgeR. We then performed nnls deconvolution of each GeoMx sample on the common genes to the target and signature matrix using the ‘nnls’ command from the nnls package.

### Likelihood and empiric *p*-value calculation for non-negative least squares metaprogram deconvolution

To assess the quality of the deconvolution using metaprograms, we compared the likelihood of the deconvolution to a shuffled null distribution of log-likelihoods under a negative binomial distribution. We first found the gene expression values that would be predicted from our deconvolution by multiplying the signature matrix by the transverse of the deconvolution matrix. This predicted gene expression matrix contained values of 0, which are not possible under a negative binomial and would give an indeterminate log-likelihood value. To account for this, we added an adjustment value to each predicted gene expression value that was equal to the smallest non-zero predicted gene expression value in the matrix. We then calculated the log-likelihood of observing the true gene expression value, rounded to the nearest whole number, under a negative binomial distribution with mu equal to the adjusted predicted gene expression value from the deconvolution. Since we had no estimate for the size parameter of the negative binomial, we calculated the log-likelihood with size parameters of 5, 10, and 20. This produced a log-likelihood for each gene in each sample. We finally summed the log-likelihood of all genes in a sample to get the total log-likelihood for each sample.

To calculate an empiric *p*-value for the deconvolution, we constructed a null distribution using a shuffling approach. We randomly shuffled the gene labels of the signature matrix, deconvoluted the data, and calculated the log-likelihood of observing the gene expression values as described above. We repeated this shuffling procedure 1000 times and thus found a null distribution of log-likelihoods for each sample. We calculated a one-tail empiric *p*-value for each sample by finding the number of null log-likelihoods that were greater than the true log-likelihoods. We report these *p*-values in Additional file 6: Table S5.

### Tumor microenvironment SpatialDecon of GeoMx data

We performed tumor microenvironment cell deconvolution of the GeoMx data using the manufacturer’s software package SpatialDecon [40]. The package contains a tumor microenvironment deconvolution signature matrix, safeTME, that is specifically designed to exclude genes that are commonly expressed in tumor cells. We further filtered this signature matrix to exclude genes that were called as variable genes in therapy-resistant WM989 and WM983b cells. Performed the deconvolution using the manufacturer’s recommended settings except for as noted below. We estimated the background from negative targeting probes using the ‘derive_GeoMx_background’ command. We then used the estimated background to perform the deconvolution using the ‘spatialdecon’ command on the upper-quantile normalized data using our filtered safeTME signature matrix (Additional file 7: Table S6) and nuclei counts from the machine with additional parameters ‘align_genes = TRUE’, ‘cellmerges = safeTME.matches’, ‘n_tumor_clusters = 5’, and ‘is_pure_tumor = grepl("S100", colnames(dsp@assayData$exprs))’. We exported the deconvolution for visualization and further analysis.

### Comparison of tumor microenvironment and metaprogram deconvolutions

Across all regions of interest, we performed pairwise correlation analysis of each resistance metaprogram against each immune cell type using the deconvolutions described above. We did so by calculating a Spearman correlation and associated *p*-value for each pair using the ‘cor’ and ‘cor.test’ functions, respectively, with parameter method = ‘spearman.’ We also employed a linear mixed-effects model using patient_id as a random intercept. *P*-values were adjusted for multiple comparisons across all program-cell type pairs using the Benjamini–Hochberg method. Associations with a false discovery rate (FDR) < 0.05 were considered statistically significant.

### Spatial transcriptomics with spatial genomics platform

*Panel design:* We designed a custom panel based on the SeqFISH technology from SpatialGenomics. The panel was chosen based on expert curation of the literature and expression in the in vitro FateMap datasets. The final panel contained 427 genes, of which 399 were detected using barcoded seqFISH imaging and 28 were identified sequentially via single-molecule FISH. The panel was designed in collaboration with SpatialGenomics and synthesized by them. The panel can be found in Additional file 9: Table S8.

*Sample preparation and imaging:* We used patient-derived xenograft (PDX) samples that had been collected in a previous study [41]. We prepared and ran a PDX sample using the manufacturer’s protocol from SpatialGenomics. We cryosectioned a 5 µm section onto a proprietary Spatial Genomics slide. We then followed the frozen tissue sample preparation method. We fixed the sample using 4% formaldehyde in PBS and then dehydrated and permeabilized with 70% ethanol. We cleared the sample with the proprietary clearing solution for 5 min, rinsed in 70% ethanol, and assembled the flow cell. We then washed the sample in the primary wash buffer, denatured primary probes at 90C for 3 min, and hybridized the primary probes to the sample at 37C overnight. The next day, we washed the sample with a primary wash buffer, stained the nuclei with a DAPI-containing staining solution, and added a rinse buffer to the sample. We then loaded the sample into the machine for rounds of secondary hybridization and readout with our custom-designed expression kit.

*Image processing and upstream analysis:* All image processing was done either on the SpatialGenomics machine or with the SpatialGenomics proprietary software. Raw images were aligned across multiple hybridizations to form composite images. Spots were detected by manually thresholding each channel to maximize spot detection while minimizing background detection. When manually thresholding, we found that a number of genes detected by smFISH were either poorly detected or had high background such that spots could not be called. We noted these nine genes (‘LDHA’, ‘ACTA2’, ‘HLA-DMA’, ‘MGP’, ‘POSTN’, ‘LUM’, ‘ITGB2’,‘CTNNB1’, ‘PECAM1’) and removed them from the analysis. The transcript identities were then decoded, and nuclei were segmented using a machine-learning algorithm on the DAPI image. A mask was formed from the segmented nuclei that included the nucleus plus a dilation around the nucleus. Individual transcripts were then assigned to individual cells, thus yielding a cell-by-gene count matrix that was exported to Python for downstream analysis.

### Spatial transcriptomics unsupervised clustering and spatial analysis

Cell-by-gene count matrices and nuclei xy-locations were imported into Python and analyzed using the Squipy package (see the accompanying.yml file for the full conda environment) [42]. One challenge was filtering out neighboring mouse cells from our analysis. To do so, we implemented multiple filtering steps prior to unsupervised analysis and clustering of cells. First, we filtered cells based on the nuclear area, only keeping those that were between 2000 and 60,000 pixels in area. These thresholds were chosen both by manual inspection of the images to compare the relative size of human and mouse cells and by looking at the distribution of cell areas. We next excluded cells that contained 50 or fewer transcripts, that contained fewer than 21 unique genes, and that contained fewer than 20 unique genes detected by barcoding rather than smFISH. Our reasoning was that mouse cells would have fewer overall transcripts and, in particular, have fewer barcoded transcripts because genes detected by the barcode method require multiple spots to be detected over multiple rounds of imaging.

We then normalized cell counts by the area of the nucleus, as has been recently recommended for this type of image-based spatial transcriptomic data [43]. We then scaled the data and performed principal component analysis (PCA). We then found neighbors using the ‘pp.neighbors’ command with parameters n_neighbors = 10 and n_pcs = 50. We then performed Uniform Manifold Approximation and Projection (UMAP) analysis using the ‘tl.umap’ command with parameter min_dist = 0.01. Finally, we performed leiden clustering using the ‘tl.leiden’ command with parameter resolution = 0.6. We found marker genes for the leiden clusters using the ‘tl.rank_genes_groups’ using both the logistic regression and Wilcoxon strategy. To quantify the relationship between xenograft Leiden clusters and the in vitro-derived metaprograms, we performed a gene overlap analysis. For each Leiden cluster identified using Scanpy's tl.rank_genes_groups function, we considered the list of the top 25 differentially expressed marker genes. For each metaprogram, we used the consensus marker gene set identified previously (see ‘Gene expression program overlap’ section and Additional file 2: Table S1 containing metaprogram definitions), filtered to include only those genes present in the 427-gene spatial transcriptomics panel. We then calculated the fraction of these available metaprogram genes that were present within the top 25 marker genes for each Leiden cluster. This overlap fraction was visualized as a heatmap (Fig. 2C). We thresholded cells as being ‘NGFR_high’ if the expression value was greater than or equal to 3, based on manual inspection of the expression distribution. Since some of these processing steps rely on random seeds, we exported these processing steps to a.h5ad file before any plots were made to maximize reproducibility. All plots were made using built-in Scanpy and Squidpy plotting functions.

## Supplementary Information

Additional file 1.

Additional file 2.

Additional file 3.

Additional file 4.

Additional file 5.

Additional file 6.

Additional file 7.

Additional file 8.

Additional file 9.

## Data Availability

All raw and processed data as well as code for analyses in this manuscript can be found at https://www.dropbox.com/scl/fo/3ftnb2a3wf7tkdepukama/AFh1uiJziyjfBBRsX47_jTI?rlkey=vvo8ctnlmjipw018xbc3odj33&dl=0 Code can also be found on Github [44] and at Zenodo [45]. Code is available under the MIT license. All raw GeoMx spatial transcriptomics used in this manuscript was from Goyal et al. and can be found at BioProject Accession PRJNA976929 [46]. All raw SeqFISH-based Spatial Genomics used in this manuscript can be found on Biostudies at S-BIAD3272 [47].

## References

[1] Shaffer SM, Dunagin MC, Torborg SR, Torre EA, Emert B, Krepler C, et al. Rare cell variability and drug-induced reprogramming as a mode of cancer drug resistance. Nature. 2017;546:431–5. 28607484 10.1038/nature22794 PMC5542814

[2] Rambow F, Rogiers A, Marin-Bejar O, Aibar S, Femel J, Dewaele M, et al. Toward Minimal Residual Disease-Directed Therapy in Melanoma. Cell. 2018;174:843-55.e19. 30017245 10.1016/j.cell.2018.06.025

[3] Roesch A, Fukunaga-Kalabis M, Schmidt EC, Zabierowski SE, Brafford PA, Vultur A, et al. A temporarily distinct subpopulation of slow-cycling melanoma cells is required for continuous tumor growth. Cell. 2010;141:583–94. 20478252 10.1016/j.cell.2010.04.020 PMC2882693

[4] Sharma SV, Lee DY, Li B, Quinlan MP, Takahashi F, Maheswaran S, et al. A chromatin-mediated reversible drug-tolerant state in cancer cell subpopulations. Cell. 2010;141:69–80. 20371346 10.1016/j.cell.2010.02.027 PMC2851638

[5] Gupta PB, Fillmore CM, Jiang G, Shapira SD, Tao K, Kuperwasser C, et al. Stochastic state transitions give rise to phenotypic equilibrium in populations of cancer cells. Cell. 2011;146:633–44. 21854987 10.1016/j.cell.2011.07.026

[6] Spencer SL, Gaudet S, Albeck JG, Burke JM, Sorger PK. Non-genetic origins of cell-to-cell variability in TRAIL-induced apoptosis. Nature. 2009;459:428–32. 19363473 10.1038/nature08012 PMC2858974

[7] Kuiken HJ, Dhakal S, Selfors LM, Friend CM, Zhang T, Callari M, et al. Clonal populations of a human TNBC model display significant functional heterogeneity and divergent growth dynamics in distinct contexts. Oncogene. 2022;41:112–24. 34703030 10.1038/s41388-021-02075-y PMC8727509

[8] Kröger C, Afeyan A, Mraz J, Eaton EN, Reinhardt F, Khodor YL, et al. Acquisition of a hybrid E/M state is essential for tumorigenicity of basal breast cancer cells. Proc Natl Acad Sci U S A. 2019;116:7353–62. 30910979 10.1073/pnas.1812876116 PMC6462070

[9] Naffar-Abu Amara S, Kuiken HJ, Selfors LM, Butler T, Leung ML, Leung CT, et al. Transient commensal clonal interactions can drive tumor metastasis. Nat Commun. 2020;11:5799. 33199705 10.1038/s41467-020-19584-1 PMC7669858

[10] Turajlic S, Sottoriva A, Graham T, Swanton C. Resolving genetic heterogeneity in cancer. Nat Rev Genet. 2019;20:404–16. 30918367 10.1038/s41576-019-0114-6

[11] Marine J-C, Dawson S-J, Dawson MA. Non-genetic mechanisms of therapeutic resistance in cancer. Nat Rev Cancer. 2020;20:743–56. 33033407 10.1038/s41568-020-00302-4

[12] Emert BL, Cote CJ, Torre EA, Dardani IP, Jiang CL, Jain N, et al. Variability within rare cell states enables multiple paths toward drug resistance. Nat Biotechnol. 2021;39:865–76. 33619394 10.1038/s41587-021-00837-3 PMC8277666

[13] Goyal Y, Busch GT, Pillai M, Li J, Boe RH, Grody EI, et al. Diverse clonal fates emerge upon drug treatment of homogeneous cancer cells. Nature. 2023;620:651–9. 37468627 10.1038/s41586-023-06342-8 PMC10628994

[14] Fennell KA, Vassiliadis D, Lam EYN, Martelotto LG, Balic JJ, Hollizeck S, et al. Non-genetic determinants of malignant clonal fitness at single-cell resolution. Nature. 2022;601:125–31. 34880496 10.1038/s41586-021-04206-7

[15] Umkehrer C, Holstein F, Formenti L, Jude J, Froussios K, Neumann T, et al. Isolating live cell clones from barcoded populations using CRISPRa-inducible reporters. Nat Biotechnol. 2021;39:174–8. 32719478 10.1038/s41587-020-0614-0 PMC7616981

[16] Oren Y, Tsabar M, Cuoco MS, Amir-Zilberstein L, Cabanos HF, Hütter J-C, et al. Cycling cancer persister cells arise from lineages with distinct programs. Nature. 2021;596:576–82. 34381210 10.1038/s41586-021-03796-6 PMC9209846

[17] Jiang CL, Goyal Y, Jain N, Wang Q, Truitt RE, Coté AJ, et al. Cell type determination for cardiac differentiation occurs soon after seeding of human-induced pluripotent stem cells. Genome Biol. 2022;23:90. 35382863 10.1186/s13059-022-02654-6 PMC8985385

[18] Reffsin S, Miller J, Ayyanathan K, Dunagin MC, Jain N, Schultz DC, et al. Single cell susceptibility to SARS-CoV-2 infection is driven by variable cell states [Internet]. bioRxiv. 2023 [cited 2024 Jun 17]. p. 2023.07.06.547955. Available from: https://www.biorxiv.org/content/10.1101/2023.07.06.547955v1. 10.1016/j.cell.2025.10.021 41240909

[19] Jain N, Goyal Y, Dunagin MC, Cote CJ, Mellis IA, Emert B, et al. Retrospective identification of cell-intrinsic factors that mark pluripotency potential in rare somatic cells. Cell Syst. 2024;15:109-33.e10. 38335955 10.1016/j.cels.2024.01.001 PMC10940218

[20] Fane ME, Chhabra Y, Alicea GM, Maranto DA, Douglass SM, Webster MR, et al. Stromal changes in the aged lung induce an emergence from melanoma dormancy. Nature. 2022;606:396–405. 10.1038/s41586-022-04774-2 PMC9554951 35650435

[21] Kaur A, Webster MR, Marchbank K, Behera R, Ndoye A, Kugel CH 3rd, et al. sFRP2 in the aged microenvironment drives melanoma metastasis and therapy resistance. Nature. 2016;532:250–4. 27042933 10.1038/nature17392 PMC4833579

[22] Kaur A, Ecker BL, Douglass SM, Kugel CH 3rd, Webster MR, Almeida FV, et al. Remodeling of the Collagen Matrix in Aging Skin Promotes Melanoma Metastasis and Affects Immune Cell Motility. Cancer Discov. 2019;9:64–81. 30279173 10.1158/2159-8290.CD-18-0193 PMC6328333

[23] Pozniak J, Pedri D, Landeloos E, Van Herck Y, Antoranz A, Karras P, et al. A TCF4/BRD4-dependent regulatory network confers cross-resistance to targeted and immune checkpoint therapy in melanoma [Internet]. bioRxiv. 2022. p. 2022.08.11.502598. Available from: https://www.biorxiv.org/content/10.1101/2022.08.11.502598v1.full.

[24] Karras P, Bordeu I, Pozniak J, Nowosad A, Pazzi C, Van Raemdonck N, et al. A cellular hierarchy in melanomauncouples growth and metastasis. Nature. 2022;610:190–8. 10.1038/s41586-022-05242-7 PMC10439739 36131018

[25] Kotliar D, Veres A, Nagy MA, Tabrizi S, Hodis E, Melton DA, et al. Identifying gene expression programs of cell-type identity and cellular activity with single-cell RNA-Seq. Elife. 2019. 10.7554/eLife.43803. 31282856 10.7554/eLife.43803 PMC6639075

[26] Barkley D, Moncada R, Pour M, Liberman DA, Dryg I, Werba G, et al. Cancer cell states recur across tumor types and form specific interactions with the tumor microenvironment. Nat Genet. 2022;54:1192–201. 35931863 10.1038/s41588-022-01141-9 PMC9886402

[27] Gavish A, Tyler M, Greenwald AC, Hoefflin R, Simkin D, Tschernichovsky R, et al. Hallmarks of transcriptional intratumour heterogeneity across a thousand tumours. Nature. 2023;618:598–606. 37258682 10.1038/s41586-023-06130-4

[28] Kinker GS, Greenwald AC, Tal R, Orlova Z, Cuoco MS, McFarland JM, et al. Pan-cancer single-cell RNA-seq identifies recurring programs of cellular heterogeneity. Nat Genet. 2020;52:1208–18. 33128048 10.1038/s41588-020-00726-6 PMC8135089

[29] Eichhoff OM, Stoffel CI, Käsler J, Briker L, Turko P, Karsai G, et al. ROS induction targets persister cancer cells with low metabolic activity in NRAS-mutated melanoma. Cancer Res. 2023;83:1128–46. 36946761 10.1158/0008-5472.CAN-22-1826

[30] Vasilevska J, Cheng PF, Lehmann J, Ramelyte E, Gómez JM, Dimitriou F, et al. Monitoring melanoma patients on treatment reveals a distinct macrophage population driving targeted therapy resistance. Cell Rep Med. 2024;5:101611. 38942020 10.1016/j.xcrm.2024.101611 PMC11293307

[31] Tsoi J, Robert L, Paraiso K, Galvan C, Sheu KM, Lay J, et al. Multi-stage differentiation defines melanoma subtypes with differential vulnerability to drug-induced iron-dependent oxidative stress. Cancer Cell. 2018;33:890-904.e5. 29657129 10.1016/j.ccell.2018.03.017 PMC5953834

[32] Benci JL, Xu B, Qiu Y, Wu TJ, Dada H, Twyman-Saint Victor C, et al. Tumor interferon signaling regulates a multigenic resistance program to immune checkpoint blockade. Cell. 2016;167:1540-54.e12. 27912061 10.1016/j.cell.2016.11.022 PMC5385895

[33] Ayers M, Lunceford J, Nebozhyn M, Murphy E, Loboda A, Kaufman DR, et al. IFN-γ-related mRNA profile predicts clinical response to PD-1 blockade. J Clin Invest. 2017;127:2930–40. 28650338 10.1172/JCI91190 PMC5531419

[34] Batlle E, Massagué J. Transforming growth factor-β signaling in immunity and cancer. Immunity. 2019;50:924–40. 30995507 10.1016/j.immuni.2019.03.024 PMC7507121

[35] Mariathasan S, Turley SJ, Nickles D, Castiglioni A, Yuen K, Wang Y, et al. TGFβ attenuates tumour response to PD-L1 blockade by contributing to exclusion of t cells. Nature. 2018;554:544–8. 29443960 10.1038/nature25501 PMC6028240

[36] Dardani I, Emert BL, Goyal Y, Jiang CL, Kaur A, Lee J, et al. ClampFISH 2.0 enables rapid, scalable amplified RNA detection in situ. Nat Methods. 2022;19:1403–10. 10.1038/s41592-022-01653-6 PMC9838136 36280724

[37] Baron M, Tagore M, Hunter MV, Kim IS, Moncada R, Yan Y, et al. The Stress-Like Cancer Cell State Is a Consistent Component of Tumorigenesis. Cell Syst. 2020;11:536-46.e7. 32910905 10.1016/j.cels.2020.08.018 PMC8027961

[38] Kinsler G, Fagan C, Li H, Kaster J, Dunne M, Vander Velde RJ, et al. SpaceBar enables clone tracing in spatial transcriptomic data [Internet]. bioRxivorg. 2025 [cited 2025 Apr 22]. p. 2025.02.10.637514. Available from: https://www.biorxiv.org/content/10.1101/2025.02.10.637514v1.abstract.

[39] Jerby-Arnon L, Shah P, Cuoco MS, Rodman C, Su M-J, Melms JC, et al. A Cancer Cell Program Promotes T Cell Exclusion and Resistance to Checkpoint Blockade. Cell. 2018;175:984-97.e24. 30388455 10.1016/j.cell.2018.09.006 PMC6410377

[40] Danaher P, Kim Y, Nelson B, Griswold M, Yang Z, Piazza E, et al. Advances in mixed cell deconvolution enable quantification of cell types in spatial transcriptomic data. Nat Commun. 2022;13:385. 35046414 10.1038/s41467-022-28020-5 PMC8770643

[41] Torre EA, Arai E, Bayatpour S, Jiang CL, Beck LE, Emert BL, et al. Genetic screening for single-cell variability modulators driving therapy resistance. Nat Genet. 2021;53:76–85. 33398196 10.1038/s41588-020-00749-z PMC7796998

[42] Palla G, Spitzer H, Klein M, Fischer D, Schaar AC, Kuemmerle LB, et al. Squidpy: a scalable framework for spatial omics analysis. Nat Methods. 2022;19:171–8. 35102346 10.1038/s41592-021-01358-2 PMC8828470

[43] Atta L, Clifton K, Anant M, Aihara G, Fan J. Gene count normalization in single-cell imaging-based spatially resolved transcriptomics. Genome Biol. 2024;25:153. 10.1186/s13059-024-03303-w PMC11167774 38867267

[44] Boe R, Raj A. Spatial transcriptomics reveals influence of microenvironment on intrinsic fates in melanoma therapy resistance. 2026. Github. https://github.com/arjunrajlaboratory/melanoma_geomx_public 10.1186/s13059-026-04112-z PMC13386816 42174697

[45] Boe R, Raj A. Spatial transcriptomics reveals influence of microenvironment on intrinsic fates in melanoma therapy resistance. 2026. Zenodo. https://zenodo.org/doi/10.5281/zenodo.19476966 10.1186/s13059-026-04112-z PMC13386816 42174697

[46] Goyal Y, Raj A. Diverse clonal fates emerge upon drug treatment of homogeneous cancer cells. Sequence Read Archive. 2023. https://www.ncbi.nlm.nih.gov/bioproject/?term=PRJNA976929 10.1038/s41586-023-06342-8 PMC10628994 37468627

[47] Boe R, Raj A. Spatial transcriptomics reveals influence of microenvironment on intrinsic fates in melanoma therapy resistance. BioStudies. 2026. https://www.ebi.ac.uk/biostudies/bioimages/studies/S-BIAD3272 10.1186/s13059-026-04112-z PMC13386816 42174697

