# Supplementary material for: Spatial transcriptomics reveals influence of microenvironment on intrinsic fates in melanoma therapy resistance
Source: Genome Biol. 2026 May 23;27:232. doi: 10.1186/s13059-026-04112-z (PMC13386816; doi:10.1186/s13059-026-04112-z)
Supplement: Supplementary file 1 — Additional file 1. [file 13059_2026_4112_MOESM1_ESM.pdf]

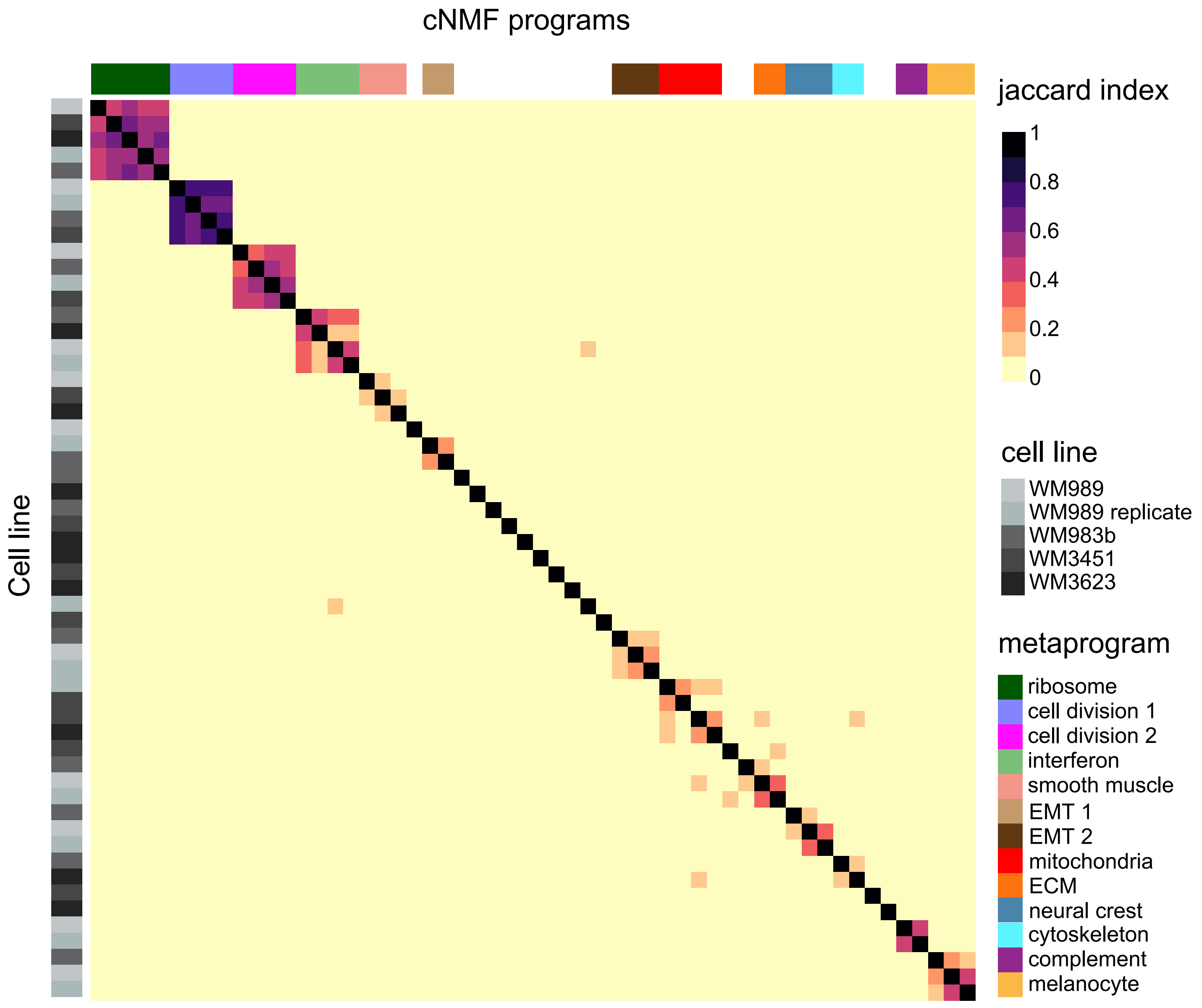

cNMF programs
Cell line
jaccard index
1
0.8
0.6
0.4
0.2
0
cell line
WM989
WM989 replicate
WM983b
WM3451
WM3623
metaprogram
ribosome
cell division 1
cell division 2
interferon
smooth muscle
EMT 1
EMT 2
mitochondria
ECM
neural crest
cytoskeleton
complement
melanocyte

Figure S1

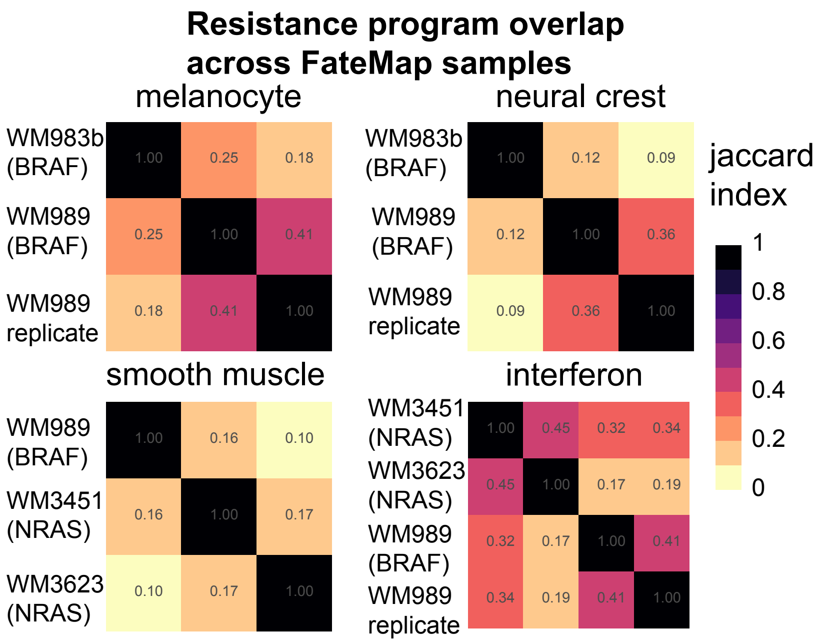
Resistance program overlap
across FateMap samples
melanocyte
neural crest
smooth muscle
interferon
jaccard index
WM983b (BRAF)
WM989 (BRAF)
WM989 replicate
WM3451 (NRAS)
WM3623 (NRAS)
1.00
0.25
0.18
0.41
0.12
0.09
0.36
0.16
0.10
0.17
0.45
0.32
0.34
0.19
1
0.8
0.6
0.4
0.2
0

Figure S2

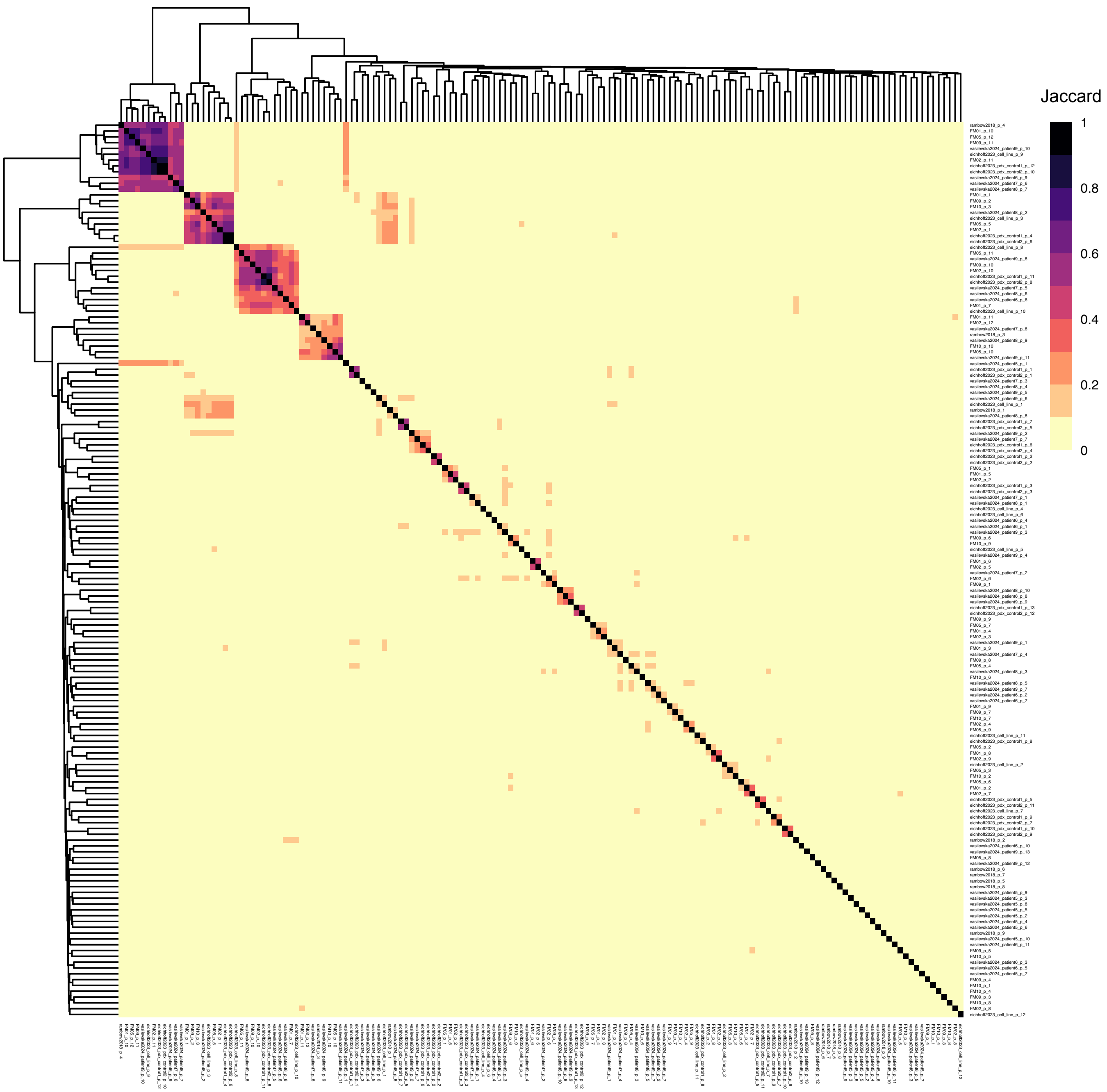

Figure S3

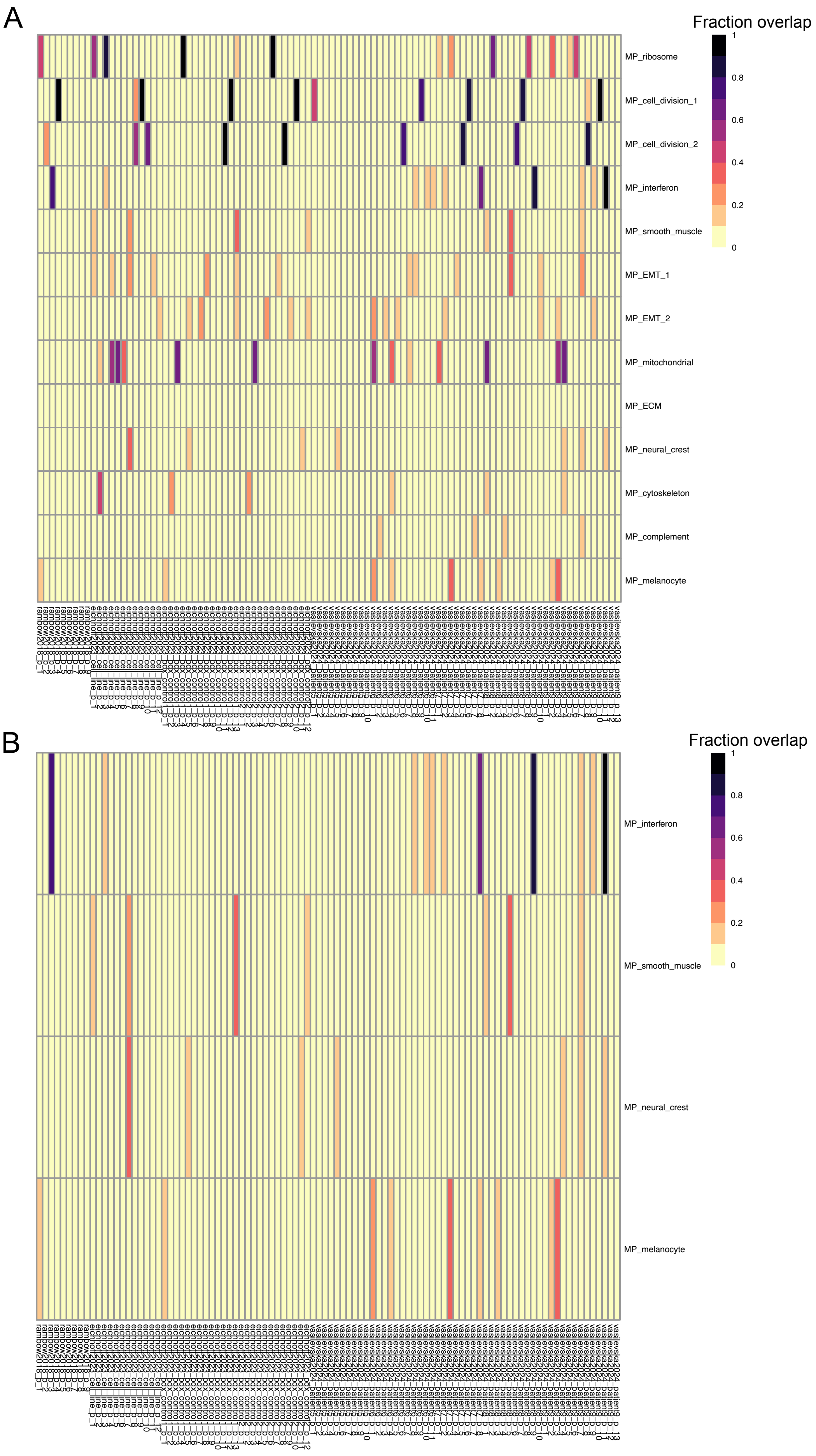
A
Fraction overlap
1
0.8
0.6
0.4
0.2
0
MP_ribosome
MP_cell_division_1
MP_cell_division_2
MP_interferon
MP_smooth_muscle
MP_EMT_1
MP_EMT_2
MP_mitochondrial
MP_ECM
MP_neural_crest
MP_cytoskeleton
MP_complement
MP_melanocyte
B
Fraction overlap
1
0.8
0.6
0.4
0.2
0
MP_interferon
MP_smooth_muscle
MP_neural_crest
MP_melanocyte

Figure S4

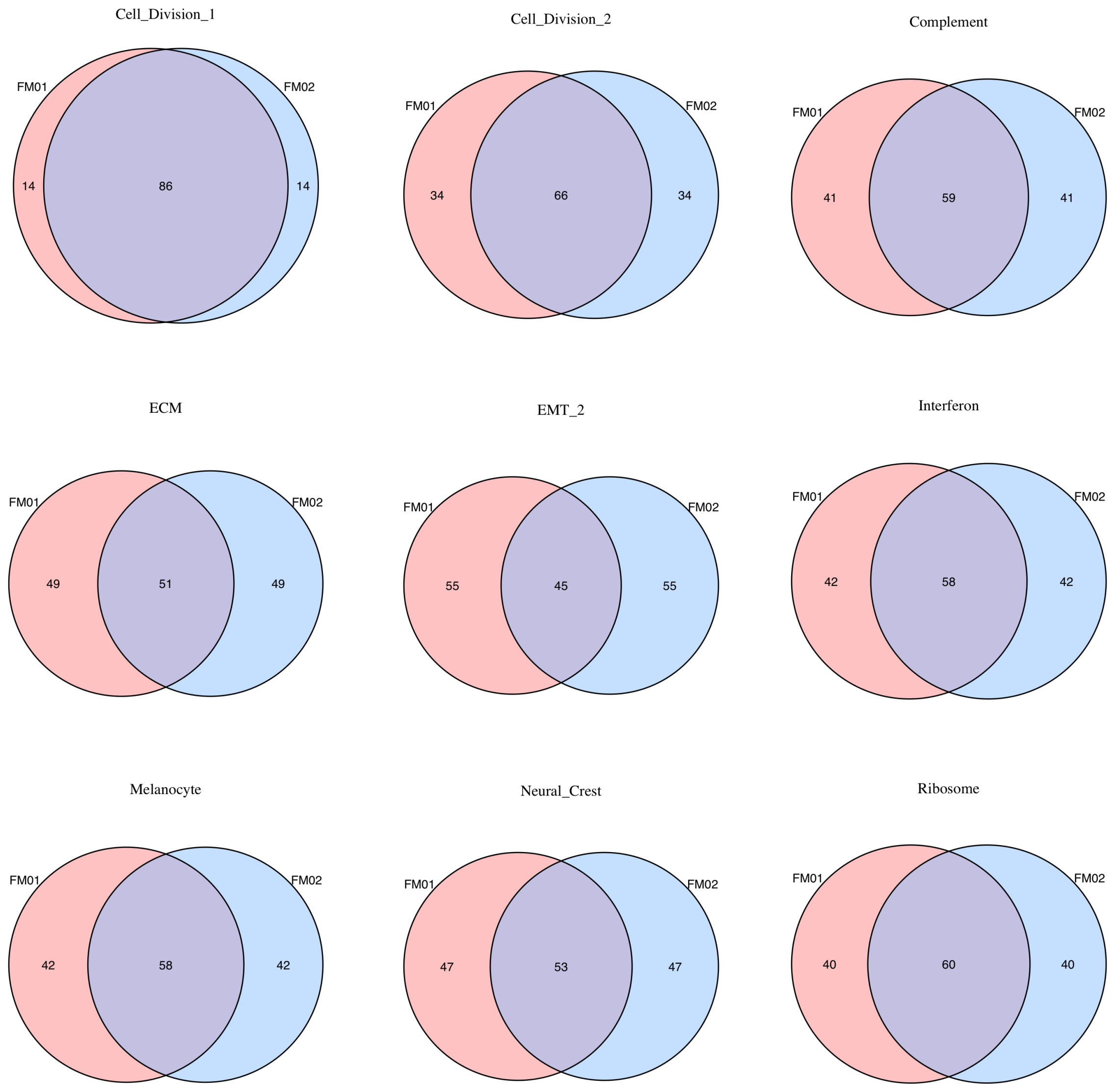

Cell_Division_1
FM01
FM02
14
86
14
Cell_Division_2
FM01
FM02
34
66
34
Complement
FM01
FM02
41
59
41
ECM
FM01
FM02
49
51
49
EMT_2
FM01
FM02
55
45
55
Interferon
FM01
FM02
42
58
42
Melanocyte
FM01
FM02
42
58
42
Neural_Crest
FM01
FM02
47
53
47
Ribosome
FM01
FM02
40
60
40

Figure S5

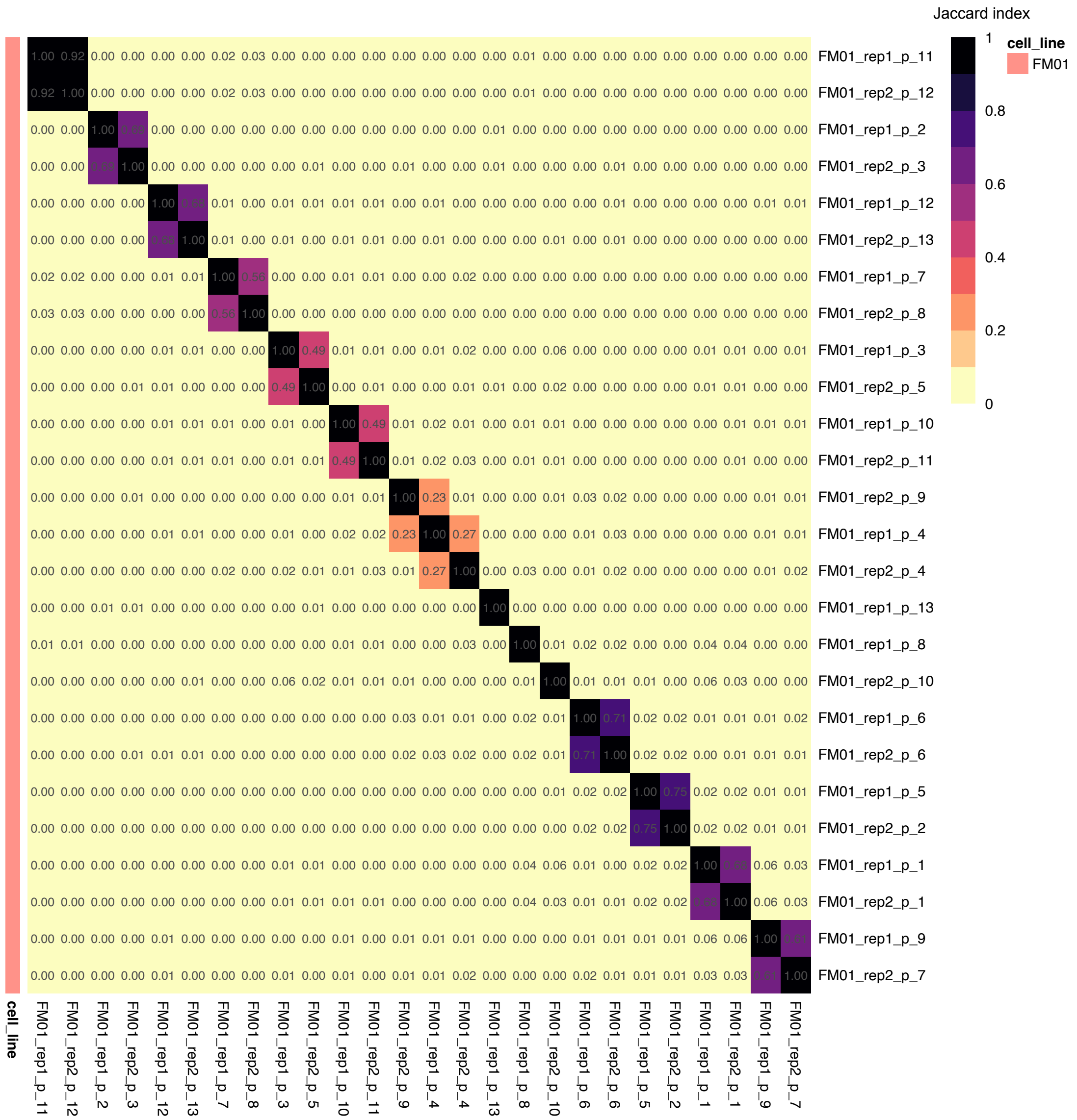
Jaccard index
1
0.8
0.6
0.4
0.2
0
cell_line
FM01
FM01_rep1_p_11
FM01_rep2_p_12
FM01_rep1_p_2
FM01_rep2_p_3
FM01_rep1_p_12
FM01_rep2_p_13
FM01_rep1_p_7
FM01_rep2_p_8
FM01_rep1_p_3
FM01_rep2_p_5
FM01_rep1_p_10
FM01_rep2_p_11
FM01_rep2_p_9
FM01_rep1_p_4
FM01_rep2_p_4
FM01_rep1_p_13
FM01_rep1_p_8
FM01_rep2_p_10
FM01_rep1_p_6
FM01_rep2_p_6
FM01_rep1_p_5
FM01_rep2_p_2
FM01_rep1_p_1
FM01_rep2_p_1
FM01_rep1_p_9
FM01_rep2_p_7
cell_line

Figure S6

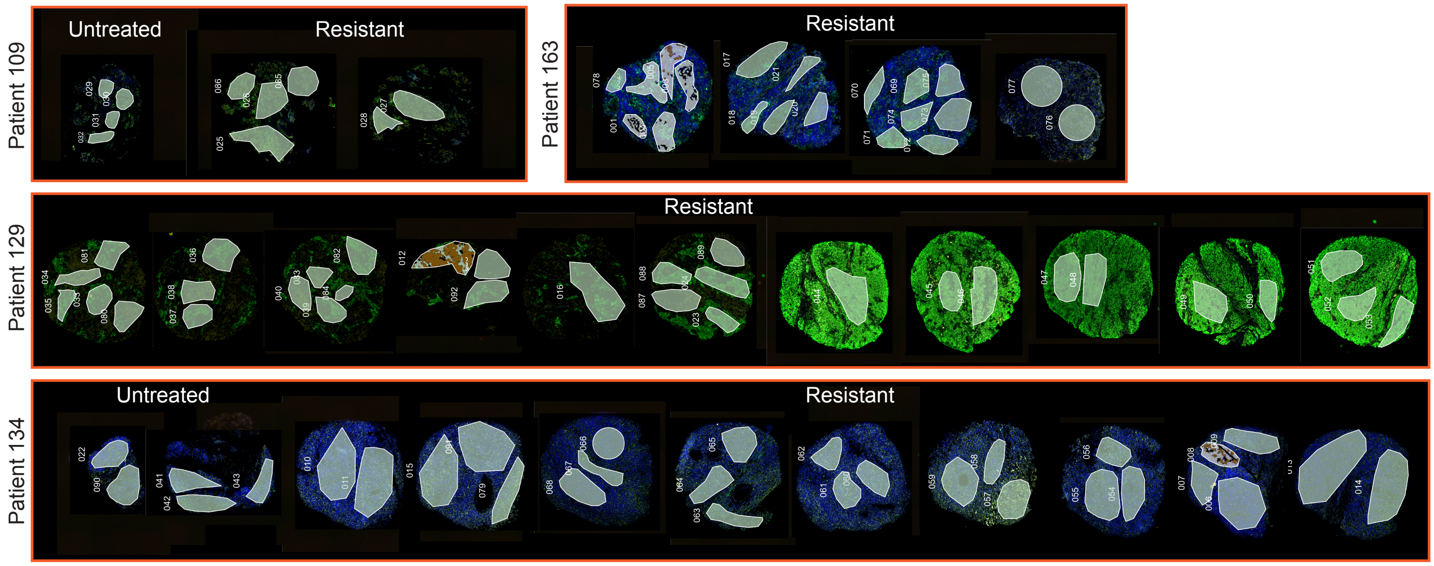
Patient 109
Untreated
Resistant
Patient 163
Resistant
Patient 129
Resistant
Patient 134
Untreated
Resistant

Figure S7

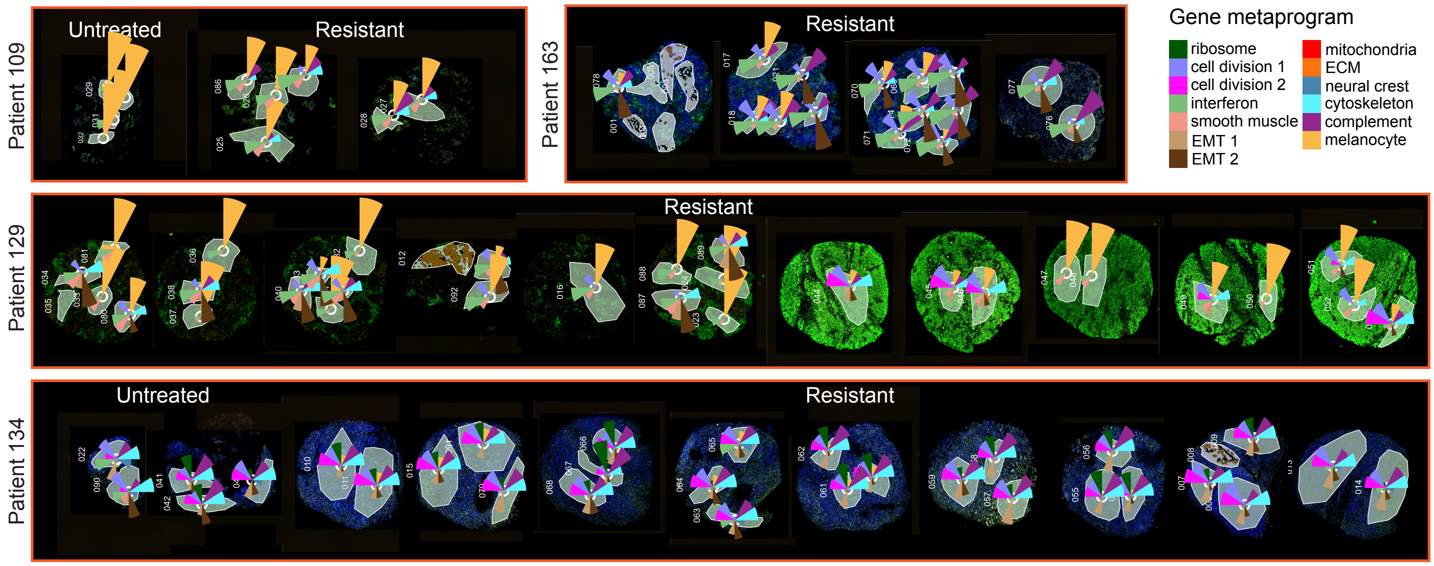
Patient 109
Untreated
Resistant
Patient 163
Resistant
Gene metaprogram
ribosome
cell division 1
cell division 2
interferon
smooth muscle
EMT 1
EMT 2
mitochondria
ECM
neural crest
cytoskeleton
complement
melanocyte
Patient 129
Resistant
Patient 134
Untreated
Resistant

Figure S8

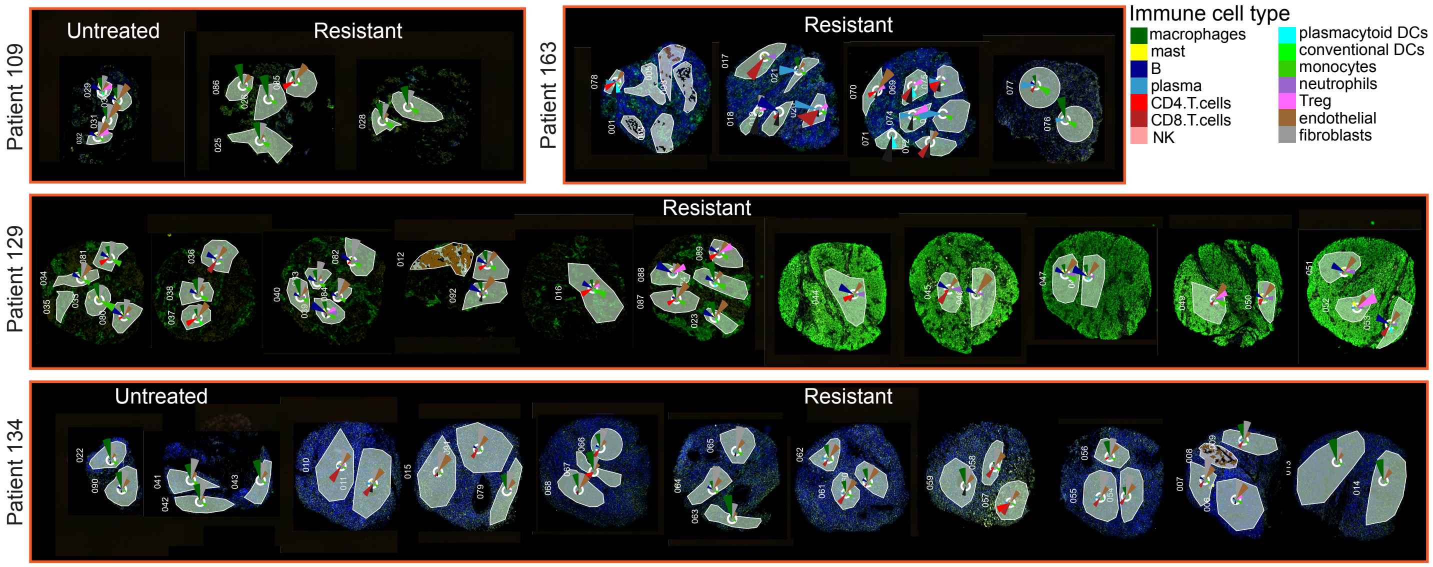
Patient 109
Untreated
Resistant
Patient 163
Resistant
Immune cell type
macrophages
mast
B
plasma
CD4.T.cells
CD8.T.cells
NK
plasmacytoid DCs
conventional DCs
monocytes
neutrophils
Treg
endothelial
fibroblasts
Patient 129
Resistant
Patient 134
Untreated
Resistant

Figure S9

A

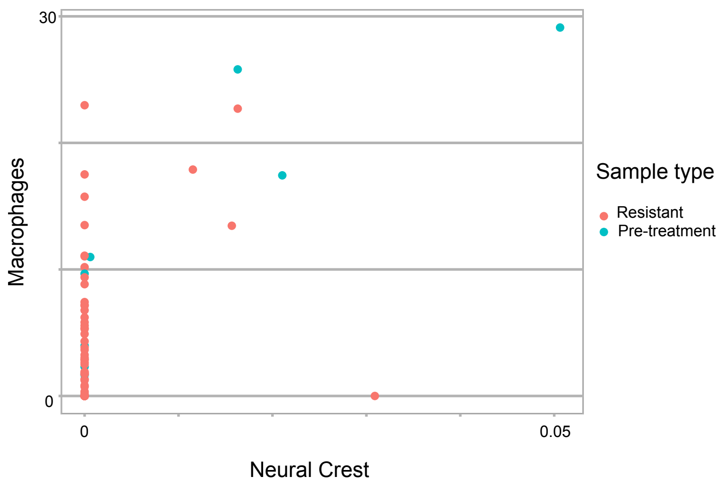

B

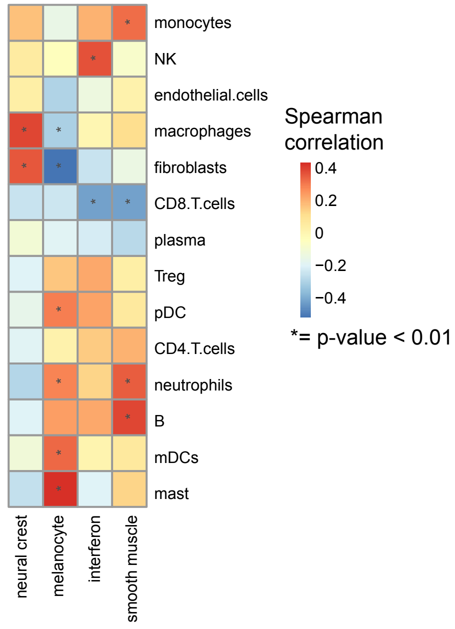

Figure S10

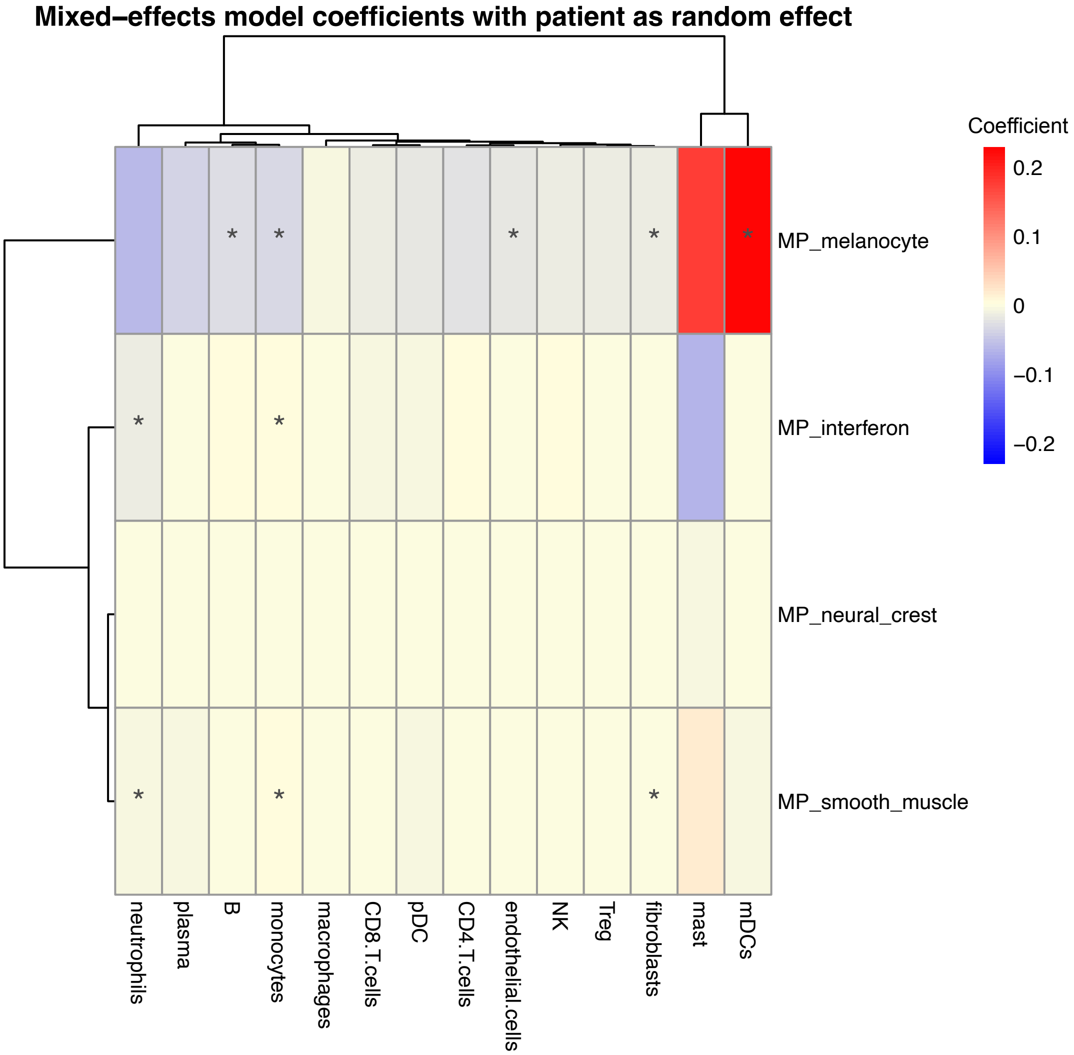

Mixed–effects model coefficients with patient as random effect
Coefficient
0.2
0.1
0
−0.1
−0.2
MP_melanocyte
MP_interferon
MP_neural_crest
MP_smooth_muscle
neutrophils
plasma
B
monocytes
macrophages
CD8.T.cells
pDC
CD4.T.cells
endothelial.cells
NK
Treg
fibroblasts
mast
mDCs

Figure S11

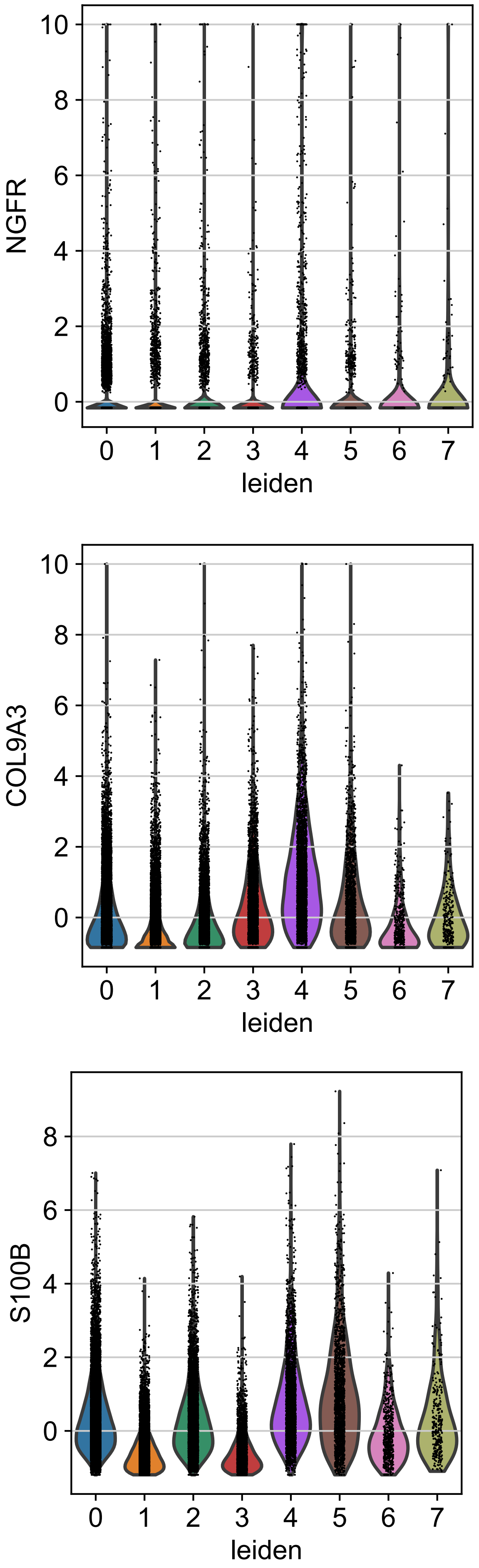

Figure S12

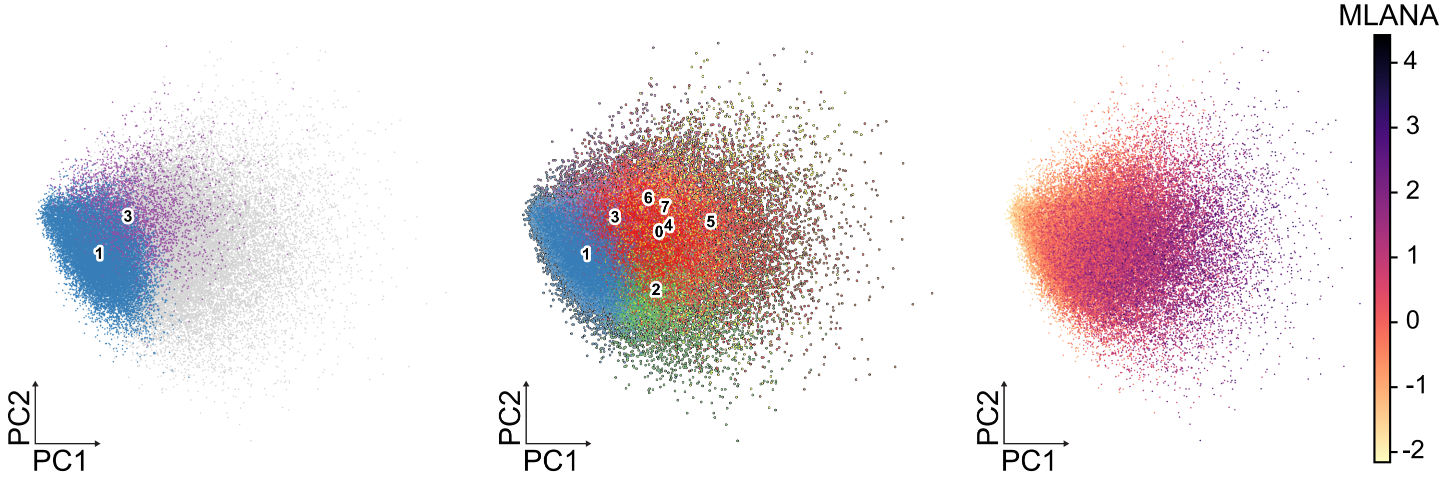

Figure S13

A IFIT2 leiden cluster 7

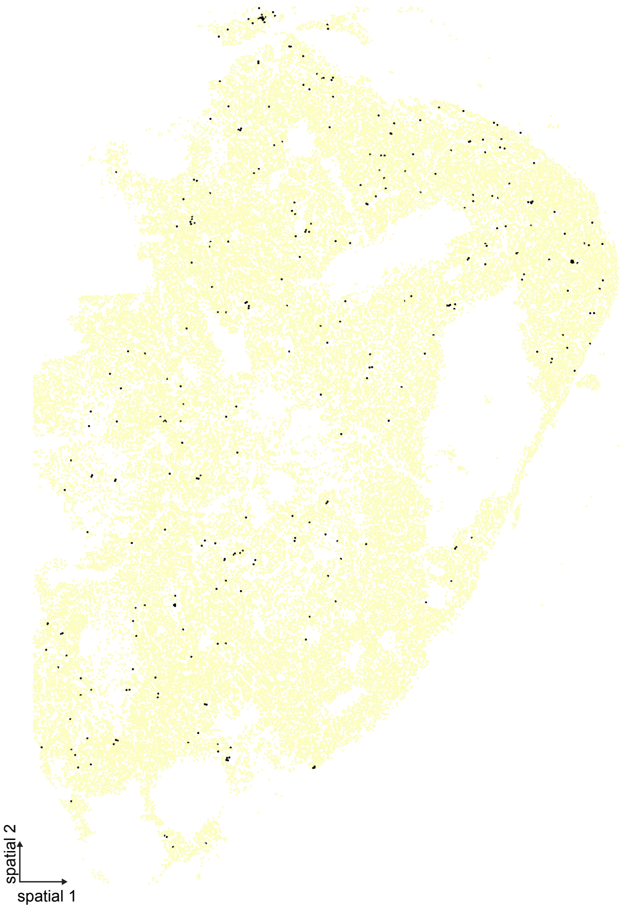

B “stress” leiden cluster 6

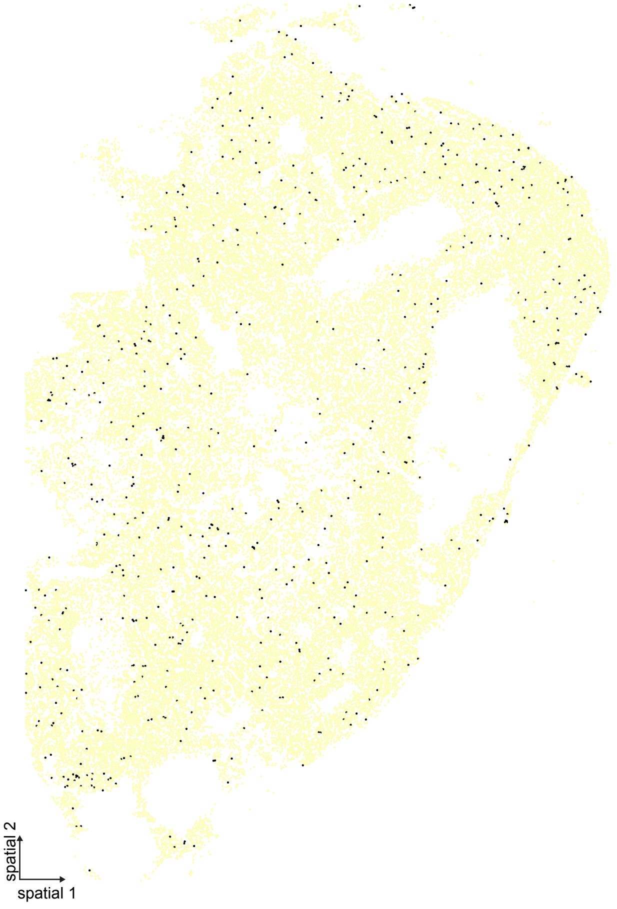

Figure S14

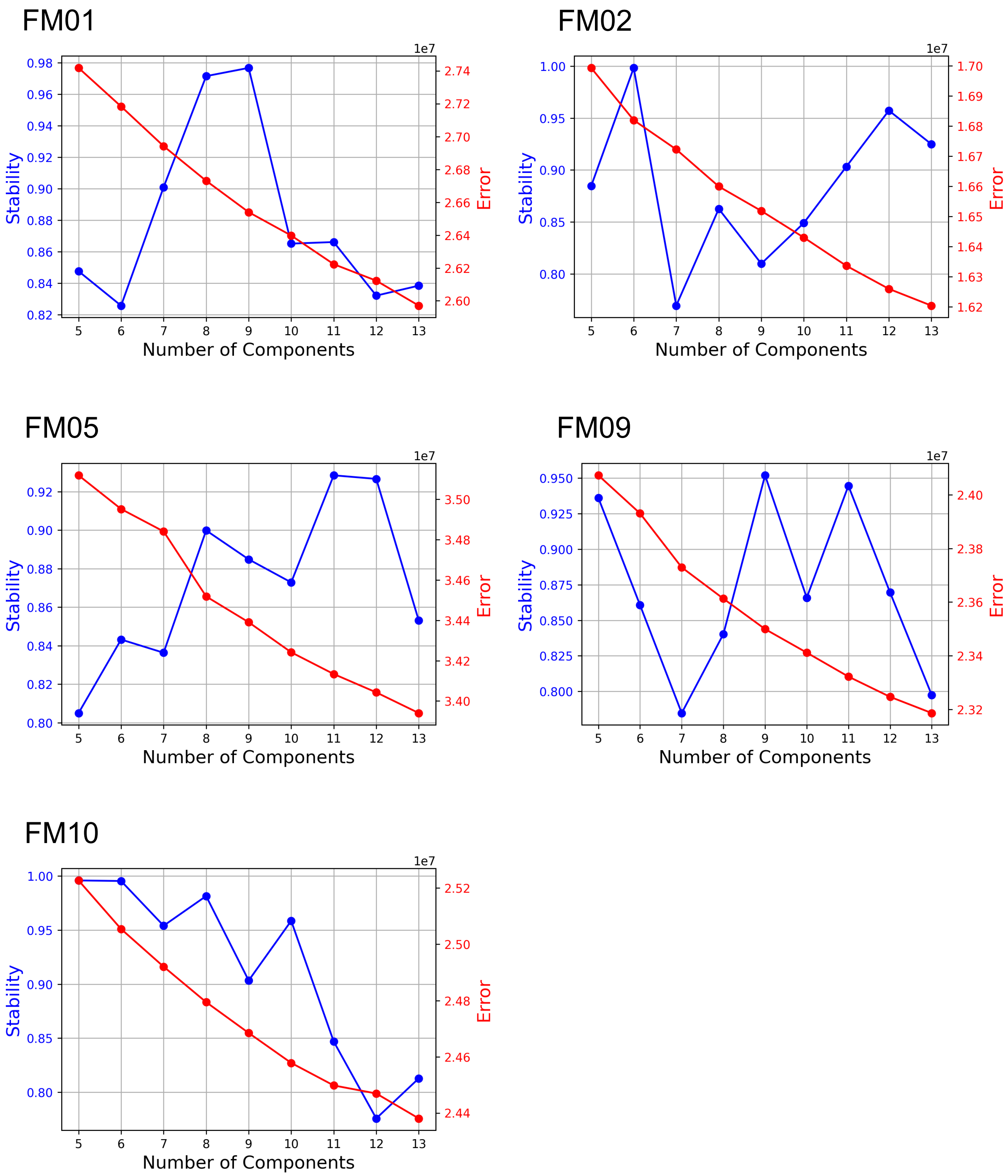
FM01
FM02
FM05
FM09
FM10
Stability
Error
1e7
Number of Components

Figure S15

FM01

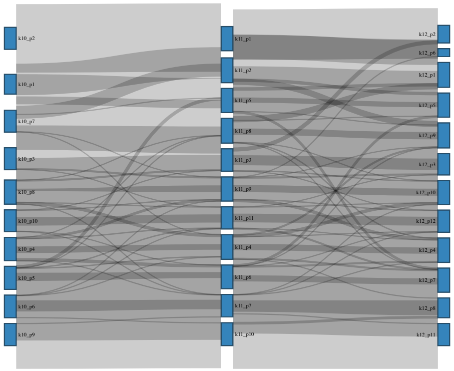

FM02

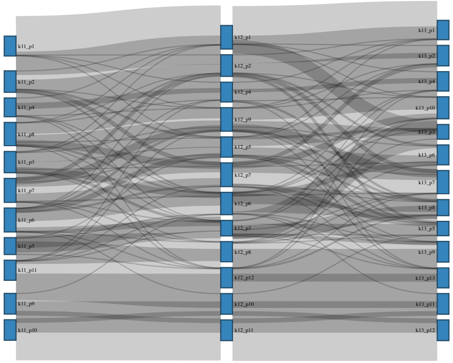

FM05

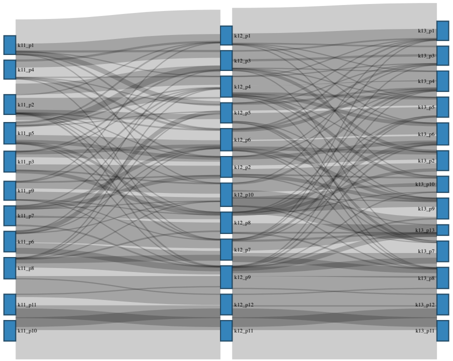

FM09

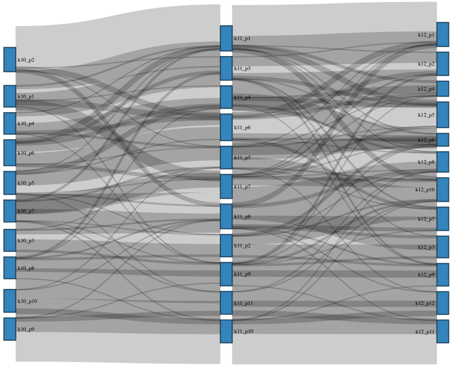

FM10

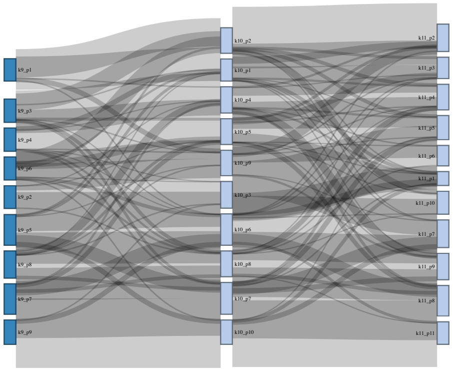

Figure S16

Supplementary figure captions

Figure S1. Heatmap of Jaccard index of top 100 marker genes for each cNMF program across all samples.

cNMF programs were identified separately for each sample. We extracted the top 100 marker genes for each program and calculated the Jaccard index between each pairwise combination of gene programs. We then performed hierarchical clustering on the results and plotted the resulting heatmap of Jaccard indices. We defined a metaprogram as a gene program that had at least a 0.1 Jaccard index between two samples. We named each metaprogram according to expert curation of the genes that appeared in all samples included in that metaprogram.

Figure S2. Heatmaps of Jaccard index for the four resistance metaprograms.

Heatmaps showing the Jaccard index for each intrinsic resistance program compared across cell lines. We identified recurrent gene programs using cNMF. We then examined the top genes for each NMF program and assigned a function to that program. We then calculated the jaccard index for the top 100 marker genes for each pairwise combination of resistance programs across all cell line samples (see supplementary figure 1 for the full heatmap). We then identified metaprograms as programs that were recurrent across multiple samples with a jaccard index of at least 0.10.

Figure S3. Heatmap of Jaccard index of top 100 marker genes for each cNMF program across all internal and external data.

cNMF programs were identified separately for each sample. We extracted the top 100 marker genes for each program and calculated the Jaccard index between each pairwise combination of gene programs. We then performed hierarchical clustering on the results and plotted the resulting heatmap of Jaccard indices.

Figure S4. Heatmaps of the fraction overlap between internally-defined metaprograms and external data cNMF programs.

Metaprograms were defined only by internal data as described previously. cNMF programs were identified separately for each external sample. The fraction overlap of each metaprogram in the top genes for each program was calculated. Panel A shows all metaprograms, whereas panel B shows only the resistance metaprograms for enhanced readability.

Figure S5. Venn diagrams of overlap between FM01 and FM02 biological replicate cNMF programs.

cNMF programs were calculated independently for FM01 and FM02 and the corresponding programs were manually identified by expert curation. The top 100 genes were pulled for each replicate and compared with Venn diagrams.

Figure S6. FM01 technical replicate Jaccard index heatmap.

cNMF programs were identified separately for each sample. We extracted the top 100 marker genes for each program and calculated the Jaccard index between each pairwise combination of gene programs. Jaccard index ranged from 0.23 - 0.92.

Figure S7. All patient sample plugs with annotated ROIs.

All 29 punch biopsies from four patients, two with matched pre-treatment samples, that were sequenced using the GeoMx Digital Spatial Profiler platform for spatial transcriptomics. 93 total ROIs were selected for sequencing based on staining for DNA (SYTO 13, blue), S100B (green), and CD45 (red).

Figure S8. Non-negative least squares deconvolution of all full GeoMx ROIs using the twelve metaprograms identified from *in vitro* cNMF analysis.

For each full ROI (that was not split based on fluorescence signal and separately sequenced), we performed non-negative least squares (nnls) deconvolution on the whole transcriptome using the expression values of twelve listed metaprograms as the signature matrix. For each ROI, we normalize the metaprogram deconvolution to one and then plot the relative amount of each metaprogram as the area of the pie wedge. The distribution of metaprogram expression shows multiple metaprograms emerge within a single patient, although each patient may have a bias towards a particular distribution of these programs.

Figure S9. Spatial deconvolution of all full GeoMx ROIs using the manufacturer's immune cell signature matrix.

For each full ROI (that was not split based on fluorescence signal and separately sequenced), we performed deconvolution on the whole transcriptome using command 'spatialdecon' from the SpatialDecon package and using the 'safeTME' immune cell signature matrix from the that we additionally filtered to remove genes that were variable genes in the WM989 and WM983b datasets to minimize the influence of transcripts from tumor cells in the estimation of immune cell amounts. For each ROI, we normalize the immune cell deconvolution to one and then plot the relative amount of each immune cell type as the area of the pie wedge.

Figure S10. Association of resistance program and immune signature including pre-treatment ROIs.

A. A small but statistically significant association between the neural crest resistance program and macrophages emerges only when including pre-treatment ROIs. Each point is an individual ROI, the x-axis is the ROI's deconvolution for the neural crest resistance fate, and the y-axis is the deconvolution for the macrophage signature. Color is whether the ROI came from a pre-treatment or resistant punch biopsy.
B. Heatmap of Spearman correlation values for resistance program compared to immune cell signature for tumor-containing ROIs. The associations change slightly when including pre-treatment samples in the analysis including a new positive correlation between the neural crest program score and the fibroblast and macrophage signature.

Figure S11. Linear mixed-effect modeling of association between resistance and immune deconvolution.

Linear mixed-effect modeling was employed with ‘patient_id’ as a random intercept. The coefficients were clustered and plotted as a heatmap. P-values were adjusted for multiple comparisons across all program-cell type pairs using the Benjamini-Hochberg method. 10 statistically-significant associations arose as compared to 11 for the Spearman method.

Figure S12. Neural crest resistant fate marker expression across leiden clusters.

Violin plots of the expression of markers of the neural crest resistant fate across unsupervised leiden clusters. *In vitro*, *NGFR*, *COL9A3*, and *S100B* are sensitive and specific markers of the neural crest resistant fate and their expression is relatively confined to a single unsupervised cluster. These three genes are enriched in cluster 4 of the PDX sample. However, unlike *in vitro*, all three genes are expressed in multiple leiden clusters, and *COL9A3* and *S100B* but not *NGFR* score as top markers of cluster 4.

Figure S13. PCA plots comparing MLANA expression.

Principal component analysis (PCA) of 51897 cells showing the first two PCs. Amount of MLANA expression, a key marker of the melanocytic state, tracks with PC1 (right plot). Clusters 1 and 3 are relatively depleted of MLANA and cluster to the left in PC1, compared to the other clusters (left and middle plots).

Figure S14. Spatial distribution of stress-like and IFIT2 clusters.

A. Spatial plot with cells in cluster 6, the “stress” cluster, as black dots and cells in all other clusters as pale dots. Cluster 6 is relatively evenly dispersed throughout the tissue but also occurs in small clusters of 3-5 cells suggesting a common parental cell of origin.
B. Spatial plot with cells in cluster 7, the IFIT2 positive cluster, as black dots and cells in all other clusters as pale dots. Like cluster 6, cluster 7 is also evenly dispersed throughout the tissue but notably occurs in clusters of 3-5 cells, again suggesting a common parental cell of origin.

Figure S15. Stability and error plots from cNMF for FM01, FM02, FM05, FM09, and FM10.

Stability and error plots generated during the cNMF algorithm for each dataset used to determine metaprograms in the main paper.

Figure S16. Alluvial plots of cNMF programs testing for robustness with k +/- 1 from values used in the main paper.

cNMF was performed individually for FM01, FM02, FM05, FM09, and FM10 using k values +/- 1 from those in the main paper. The top 100 genes for each program were pulled and alluvial plots generated for each dataset. cNMF programs were largely stable between different k values, indicating robustness of the procedure.
